# CT-based automatic segmentation of key CSF regions for detecting disproportionately enlarged subarachnoid space hydrocephalus

**DOI:** 10.1186/s12987-026-00814-5

**Published:** 2026-06-23

**Authors:** Shigeki Yamada, Hirotaka Ito, Keisuke Hagiwara, Yasuo Kawata, Chifumi Iseki, Motoki Tanikawa, Tomohiro Otani, Satoshi Ii, Yoshiyuki Watanabe, Shigeo Wada, Marie Oshima, Mitsuhito Mase

**Affiliations:** 1 Department of Neurosurgery, Nagoya City University Graduate School of Medical Sciences, Kawasumi 1, Mizuho-cho, Mizuho-ku Nagoya, Aichi, 467-8601 Japan; 2 Interfaculty Initiative in Information Studies/Institute of Industrial Science, The University of Tokyo, Tokyo, Japan; 3 Medical System Research & Development Center, FUJIFILM Corporation, Tokyo, Japan; 4 Department of Behavioral Neurology and Cognitive Neuroscience, Tohoku University Graduate School of Medicine, Sendai, Miyagi Japan; 5 Division of Neurology and Clinical Neuroscience, Department of Internal Medicine III, Yamagata University School of Medicine, Yamagata, Japan; 6 Department of Mechanical Science and Bioengineering, Graduate School of Engineering Science, The University of Osaka, Osaka, Japan; 7 Department of Mechanical Engineering, School of Engineering, Institute of Science Tokyo, Tokyo, Japan; 8 Department of Radiology, Shiga University of Medical Science, Shiga, Japan

**Keywords:** Chronic hydrocephalus, Disproportionately enlarged subarachnoid-space hydrocephalus, DESH, Hakim’s disease, Idiopathic normal pressure hydrocephalus, Computed tomography (CT), Artificial intelligence, Cycle-GAN, 3D U-Net model, Semantic segmentation

## Abstract

**Background:**

Disproportionately enlarged subarachnoid space hydrocephalus (DESH) is a characteristic neuroimaging feature of idiopathic normal pressure hydrocephalus (iNPH), a treatable but frequently underdiagnosed condition. While brain MRI enables detailed region-based segmentation, CT is faster and widely used in daily clinical practice. However, objective and automated evaluation of DESH-related features on plain CT remains challenging. This study aimed to develop and validate an artificial intelligence-based segmentation model for automatically extracting four cerebrospinal fluid (CSF) regions from plain CT.

**Methods:**

Synthetic CT images were generated from annotated 3D T1-weighted MRI using a cycle-generative adversarial network (Cycle-GAN) to create training data. However, segmentation accuracy based on synthetic CT was insufficient. therefore, the final model was trained using manually annotated real CT images. A deep learning model was developed to segment four CSF regions (total ventricles, total subarachnoid space [SAS], high-convexity SAS, and Sylvian fissures with basal cisterns). Model performance was evaluated using an external validation dataset (30 DESH and 30 non-DESH). In addition, a second independent validation set consisting of 115 consecutive patients was used to assess clinical utility and determine optimal cutoff values using receiver operating characteristic analysis.

**Results:**

Based on external validation Dice scores, Version 10 was adopted as the final model. In internal validation, the final model achieved Dice scores > 0.9 for total ventricles and > 0.8 for total intracranial CSF space, with lower scores for high-convexity SAS and Sylvian fissures with basal cisterns. External validation yielded Dice scores of 0.92, 0.85, 0.60, and 0.94 for the four regions, respectively. In the second validation set, the DESH index demonstrated excellent diagnostic performance, with an area under the curve of 1.00 and an optimal cutoff value of 10 (sensitivity 100%, specificity 100%). Venthi and Sylhi indices also showed high diagnostic performance.

**Conclusions:**

The proposed artificial intelligence model enables fully automated segmentation and quantitative assessment of DESH-related CSF regions on plain CT with high reliability. This approach may improve diagnostic accuracy, facilitate earlier detection, and support objective evaluation of postoperative changes in iNPH. Its applicability to routine CT makes it particularly valuable in settings where MRI is unavailable or contraindicated.

**Supplementary Information:**

The online version contains supplementary material available at 10.1186/s12987-026-00814-5.

## Background

Recently, there has been growing attention to normal pressure hydrocephalus (NPH), a neurological disorder characterized by the classic triad of gait disturbance, cognitive impairment, and urinary incontinence in the elderly [1–3]. NPH is typically generally classified into idiopathic NPH (iNPH) and secondary NPH (sNPH), the latter of which develops following conditions such as subarachnoid hemorrhage, traumatic brain injury, or meningitis [2, 3], In the context of a super-aging society, the prevalence of iNPH is expected to increase substantially. However, it is estimated that fewer than 10% of affected individuals receive an accurate diagnosis, and an even smaller proportion undergo appropriate treatment [4–9]. To overcome the current situation in which many patients remain undiagnosed, the task force of the International Hydrocephalus Society undertook a review of the classification and terminology related to NPH. Finally, they proposed renaming NPH to chronic hydrocephalus in adults (CHiA) and iNPH to Hakim’s disease [10]. Another reason for the low diagnostic rate is that iNPH is frequently misdiagnosed as cerebral atrophy, even when head CT or MRI is performed. This is because its imaging features differ from those of hydrocephalus of genetic or congenital etiology traditionally diagnosed in clinical practice [11]. In iNPH, the ventricles are not markedly enlarged compared to those in the other type of chronic hydrocephalus in adults; instead, there is disproportionate enlargement of the Sylvian fissures accompanied by a simultaneous narrow sulci in the high-convexity region [11–15]. These characteristic imaging findings are increasingly recognized as disproportionately enlarged subarachnoid-space hydrocephalus (DESH) [16–20]. To support the automated diagnosis of iNPH, we developed an artificial intelligence-based computer-aided diagnosis (AI-CAD) system in 2024 [21], which detects DESH by extracting the relevant volumes of interest (VOIs) from 3D T1 or T2-weighted MRI [22]. However, given the limitations of 3D MRI in terms of accessibility and cost, there is a growing need for more practical alternatives. Plain CT, which is more commonly used in clinical practice, offers several advantages, including shorter scan durations and greater cost-effectiveness. For instance, when elderly individuals sustain a head injury due to a fall, exhibit gait and balance disturbances, or present with cognitive impairments, they are often evaluated with a head CT scan. However, a persistent challenge is that DESH may frequently be overlooked on CT images, particularly in the absence of overt ventricular enlargement, leading to missed opportunities for early diagnosis and intervention. Developing an AI-CAD system capable of detecting DESH on standard head CT images could therefore provide substantial clinical benefit by enabling early identification of iNPH, even in patients undergoing imaging for unrelated indications. Nonetheless, the lack of anatomical labels in CT datasets presents a major obstacle to AI. Therefore, the aim of this study was to develop a CT-based AI-CAD system for the automated diagnosis of DESH. To achieve this, we employed a two-step strategy. First, we generated synthetic CT data from annotated 3D MRI using cycle-generative adversarial networks (cycle-GAN) [23] followed by manual refinement, wherein the synthetic CT images and corresponding transferred labels were reviewed and corrected by experts to ensure anatomical validity prior to model training [21]. Second, we trained a 3D U-Net model using the refined CT data to enable robust and accurate segmentation of DESH-related features directly from real CT images.

## Methods

### Study population

The dataset of plain head CT images in this study included patients from the four collaborating hospitals. CT data were retrospectively collected from January 2023 to December 2024 from consecutive patients who underwent head CT in neurosurgery or neurology inpatient and outpatient settings. Informed consent was waived, and an opt-out approach was applied in accordance with institutional review board approval. The inclusion criteria for patients were individuals aged 60 years or older who underwent head CT for any reason. The diagnosis of iNPH was established based on the presence of DESH and at least one symptom of Hakim’s triad, in accordance with the third edition of the Japanese guidelines [3]. CT scans of patients with iNPH included not only preoperative images but also postoperative ones. The exclusion criteria were patients with chronic hydrocephalus in adults in any of the six categories other than iNPH, such as those with severe ventriculomegaly characteristic of midlife hydrocephalus [10], and those with CT scans showing space-occupying intracranial lesions such as intracranial hemorrhage or brain tumors. Three CT data were excluded, because two were incidentally found to have chronic subdural hematomas, and one was found to have a brain tumor.

### Data preparation and deep learning methods

**Fig. 1:**
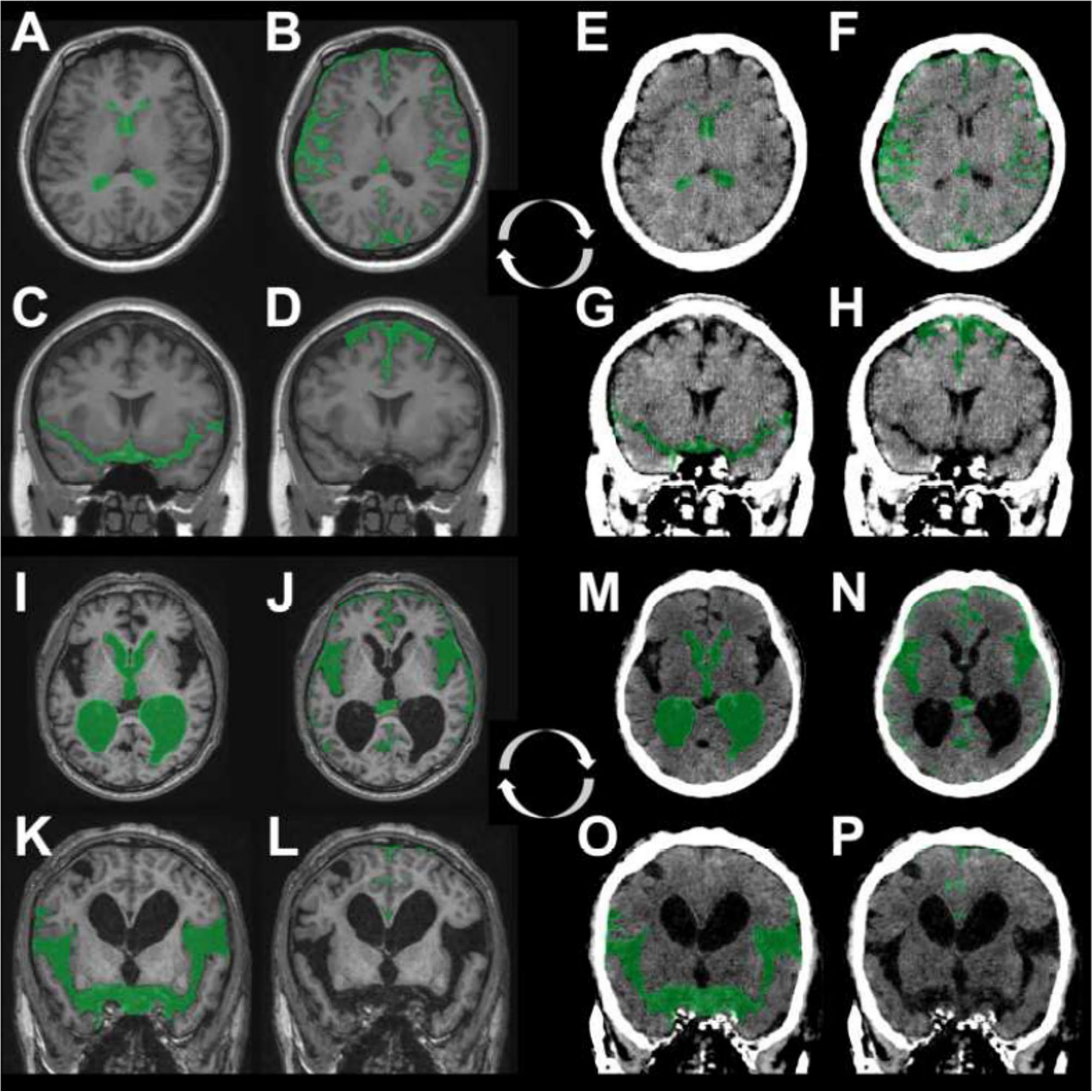
Synthetic CT Generated from 3D MRI in a Healthy Individual and a Patient with iNPH. **A–H**: healthy individual. **A**: total ventricles, **B**: total subarachnoid space, **C**: Sylvian fissures and basal cisterns, **D**: high-convexity subarachnoid space. Green-colored overlays indicate the segmented regions of interest on the original 3D MRI images. **E–H**: corresponding synthetic CT images generated from A–D using Cycle-GAN. **I–P**: patient with iNPH. **I**: total ventricles, **J**: total subarachnoid space, **K**: Sylvian fissures and basal cisterns, **L**: high-convexity subarachnoid space. Green-colored overlays indicate the segmented regions of interest on the original 3D MRI images. **M–P**: corresponding synthetic CT images generated from I–L using Cycle-GAN

As a first step in developing a deep learning model, synthetic CT images (matrix 512 × 512; voxel size, 0.9 × 0.9 × 0.9 mm) were generated using cycle-GAN from T1-weighted 3D magnetization-prepared rapid gradient echo (MPRAGE) images (matrix 256 × 256; voxel size, 0.9 × 0.9 × 0.9 mm), which had previously been annotated with masks for the total ventricles, total subarachnoid spaces (SAS), high-convexity SAS, and a combined region of the Sylvian fissure and basal cistern (Fig. 1) [21]. A total of 125 annotated synthetic CT scans were prepared by directly transferring the MRI annotations onto the corresponding synthetic CT images (Additional file 1).

In the second step, these annotated synthetic CT scans were used as training data for a semantic segmentation task using deep learning with a four-layer 3D U-Net, following the same model architecture as in our previous study using the SYNAPSE Creative Space cloud-based AI development platform (FUJIFILM Corporation) [21]. Briefly, the model consisted of 3D convolutional layers with batch normalization, ReLU activation, max pooling, and 3D up-convolutional layers. Signal intensities were normalized by percentile (minimum 0.05, maximum 0.95) as a preprocessing step. To preserve voxel-level detail, feature maps extracted at each encoding layer were concatenated with the corresponding decoding layers via skip connections between the downsampling and upsampling paths. In addition, data augmentations including rotation, scaling, and translation of the input image masks were applied to improve the generalizability and accuracy of the semantic segmentation. These augmentations were expected to reduce the impact of variations across manufacturers, imaging protocols, and individual differences, thereby increasing the robustness of the AI model.

1. *Accuracy Optimization Phase (Versions 1–7)*

**Fig. 2:**
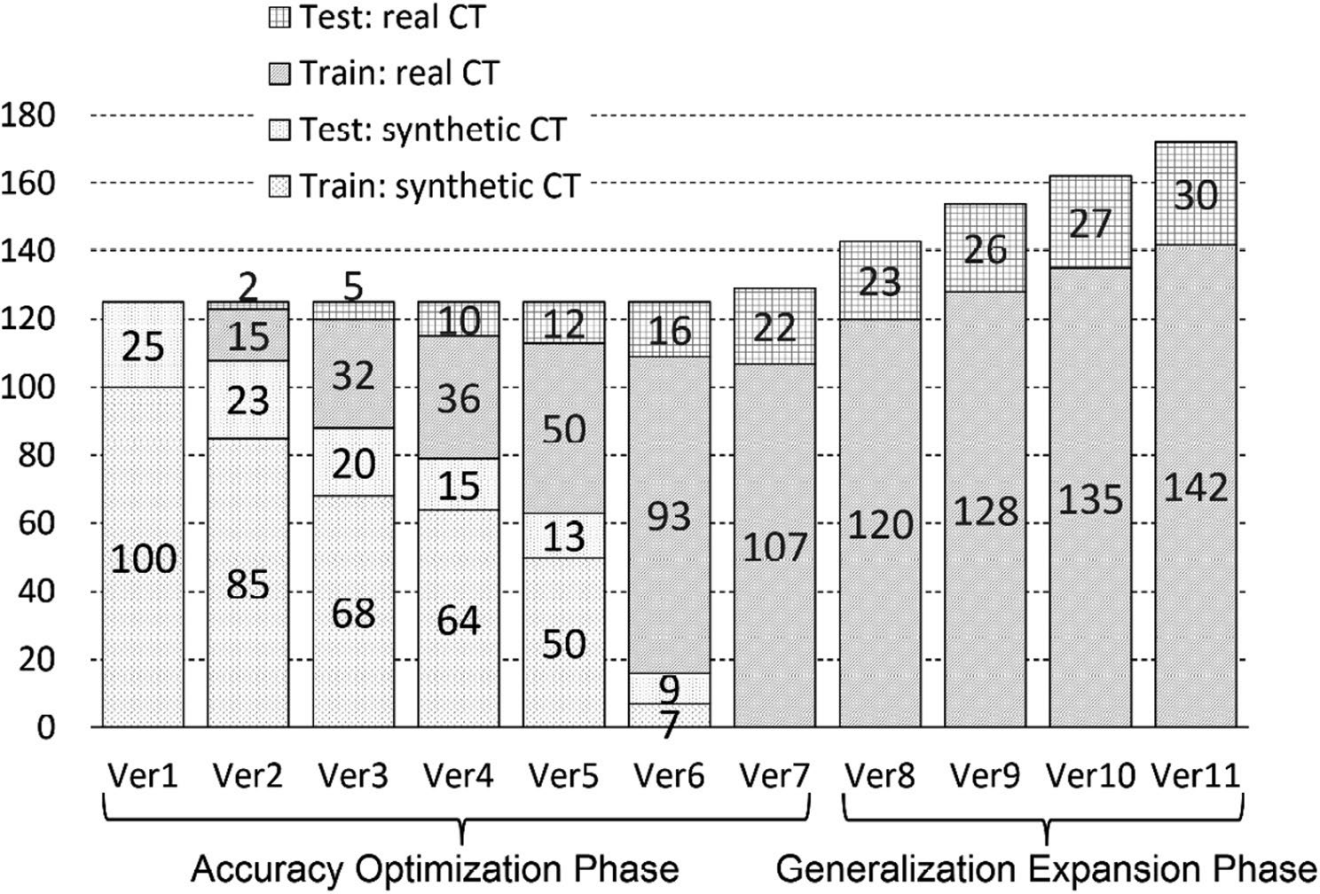
Number of synthetic and real CT datasets used for AI model training and internal validation across versions. The figure illustrates the number of synthetic CT images generated from annotated 3D T1-weighted MRI using cycle-GAN and real CT images used for training and internal validation of each AI model version. Train: synthetic CT refers to the number of synthetic CT datasets used to train the initial models (Versions 1–6). Test: synthetic CT indicates the synthetic CT images used for internal validation during the early versions. Train: real CT represents the manually annotated real CT datasets incorporated into training from Version 2 onward, gradually replacing synthetic CTs. Test: real CT refers to real CT datasets used for internal validation throughout all versions. By Version 7, all synthetic CT datasets had been fully replaced with real CT datasets for training

The initial AI model (Version 1) was trained for a semantic segmentation task using only 125 synthetic CT images (Figs. 2). This first-generation AI model was then applied to 17 real CT images (matrix 512 × 512; slice thickness: 0.5–1.0 mm) to automatically extract four VOIs. These extracted VOIs were manually corrected by the same expert (S.Y.) who had previously annotated the MRI data [21] (Additional file 2). These corrected masks were used as annotations and, along with the corresponding real CT images, replaced an equal number of synthetic CT images. A new 3D U-Net model was then trained from scratch, resulting in the second-generation AI model (Version 2). Using Version 2, the same procedure—automatic extraction followed by manual correction—was performed on 20 real CT images (matrix 512 × 512; slice thickness: 0.45–2.0 mm). Newly acquired real CT data were incorporated into the training set, with an equal number of synthetic CT images removed to develop the next version of the AI model. This iterative process continued, and by the development of the seventh-generation model (Version 7), all synthetic CT images had been replaced with real CT data manually annotated with expert-defined VOIs, resulting in output that required minimal manual correction (Fig. 2).

2) *Generalization Expansion Phase (Versions 8–11)*

Beginning with Version 8, the training dataset was expanded to include CT images with a slice thickness of 5.0 mm and those depicting intraventricular catheters placed in the lateral ventricles following ventriculoperitoneal shunt (VPS) surgery. Ventricular catheter structures were explicitly excluded, and only catheter-free CSF regions were used as ground truth for model training, enabling the model to avoid including catheter structures in ventricular and SAS segmentation. The same training procedure was repeated with the increasing number of real CT images, aiming to broaden the generalizability of automatic segmentation rather than to further improve accuracy, through Version 11 (Fig. 2).

### Validation study

For external validation, we used CT images from four collaborating hospitals, excluding those used for training and internal validation of the 3D U-Net model. The first validation dataset consisted of images from 30 patients diagnosed with DESH (including 25 with iNPH) and 30 patients who underwent head CT for other reasons, such as head injury, and were confirmed to be free of DESH. DESH classification was independently performed by two experienced experts (S.Y. and Y.W.), both involved in the development of MRI-based DESH analysis, and final classification was determined by consensus. The VOIs of the total ventricles, high-convexity SAS, Sylvian fissure and basal cistern, and total intracranial cerebrospinal fluid (CSF) space extracted by AI models from Version 7 to Version 11 were evaluated against the ground truth masks using the Dice score.

In addition, to further evaluate the clinical utility of the developed AI segmentation application and to establish thresholds for the derived indices, a second validation set was collected at a collaborating institution. Plain head CT data were retrospectively collected between December 2025 and January 2026 from consecutive patients aged ≥ 20 years. The indication for CT was unknown. Cases with subarachnoid hemorrhage, intracranial hemorrhage, or those obtained immediately after craniotomy were excluded. A total of 115 patients were included in the final analysis (56 males, 59 females; mean age, 77.5 ± 13.3 years).

### Three-dimensional volumetric index

The “DESH index” was defined as the combined volume of the total ventricles and Sylvian fissure and basal cistern divided by the high-convexity part of the subarachnoid space volume, as previously proposed in our development of an MRI-based AI-CAD system [21]. In relation to the supplemental indices for DESH, the “Venthi index” was defined as the total ventricular volume divided by the high-convexity part of the subarachnoid space volume, while the “Sylhi index” was defined as the volume of the Sylvian fissure and basal cistern divided by the high-convexity part of the subarachnoid space volume.

### Statistical analysis

Mean age, segmented volumes, and the three indices were compared between patients with DESH and elderly patients without DESH using the Mann–Whitney–Wilcoxon test. For patients with iNPH whose pre- and postoperative CT scans were included in the external validation, the extracted VOIs and three indices were compared between the pre- and postoperative scans using a paired t-test. To quantify the performance, e.g., the accuracy and similarity of the volumetric semantic segmentation, the Dice coefficient score for the loss function was calculated as 2 * |X ∩ Y| + epsilon(1e-4) / (|X| + |Y| + epsilon(1e-4)) in the validation study. X and Y were the prediction and correct, binary [0, 1] output per voxel. The area under the receiver-operating characteristic curves (AUCs) and optimal thresholds for detecting DESH, ventriculomegaly, THC, and SFD were calculated to maximize the sum of the sensitivities and specificities by maximizing Youden’s Index. Using the optimal thresholds from AUC analyses, the odds ratios (ORs) with 95% confidential intervals (CIs) using thresholds were calculated. All missing variables were considered as deficit data, and no other variables were adjusted. Statistical significance was set at a probability value (*P*) of < 0.05. All statistical analyses were performed using the R software (version 4.4.0, R Foundation for Statistical Computing, Vienna, Austria, http://www.R-project.org).

## Results

### Segmentation accuracy in internal and external validation

**Fig. 3:**
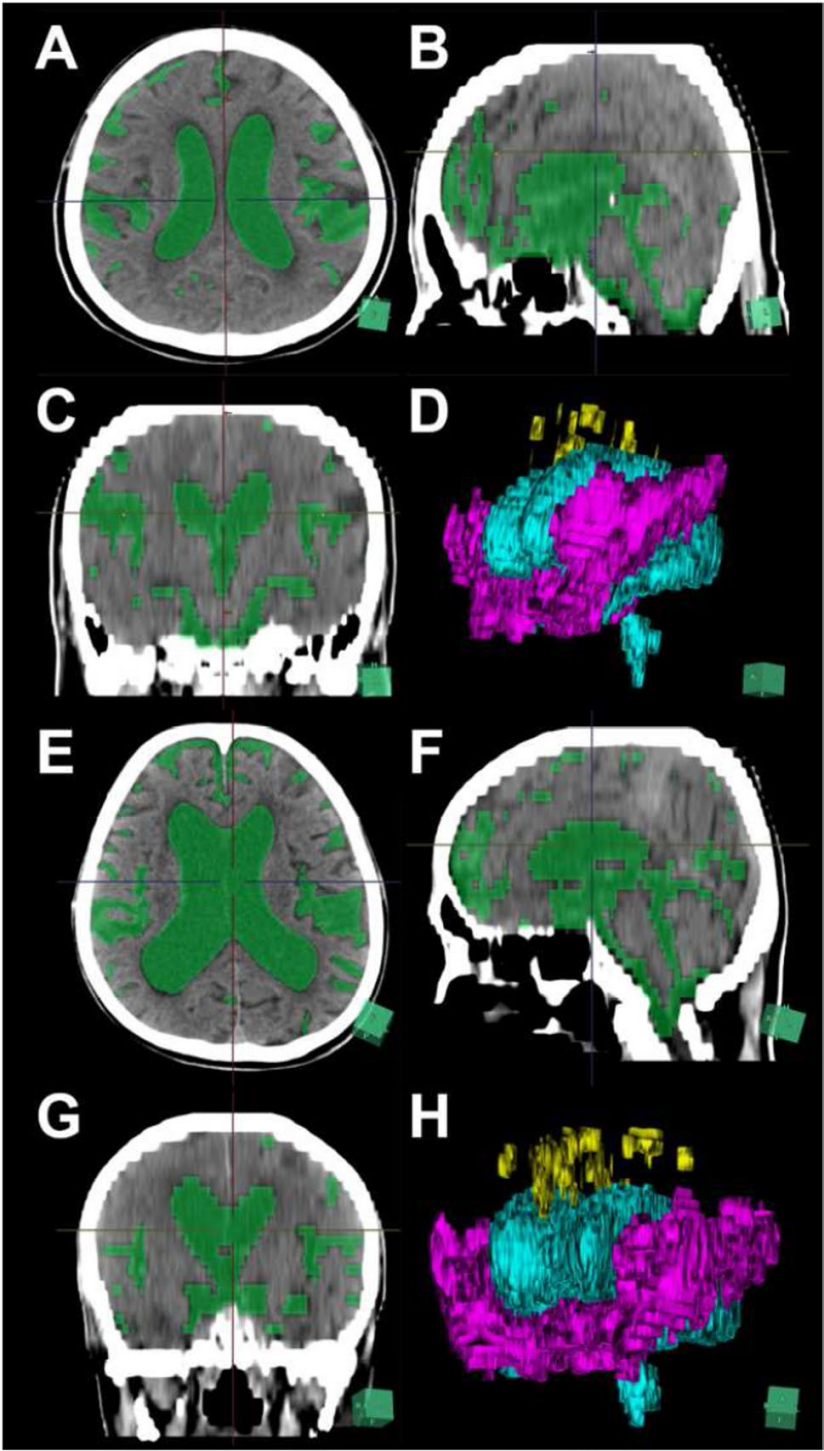
Performance of AI models from version 7 to version 9 on 5.0-mm slice thickness CT scans. Segmentation masks were generated from plain CT images with a slice thickness of 5.0 mm in an 80-year-old male patient with iNPH using AI models from Version 7 (**A–D**) to Version 9 (**E–H**). The segmented regions include: **A–C**,** E–G**: Total intracranial CSF space (green); **D**,** H**: Total ventricles (sky blue), high-convexity SAS (yellow), and Sylvian fissures with basal cisterns (magenta)

**Fig. 4:**
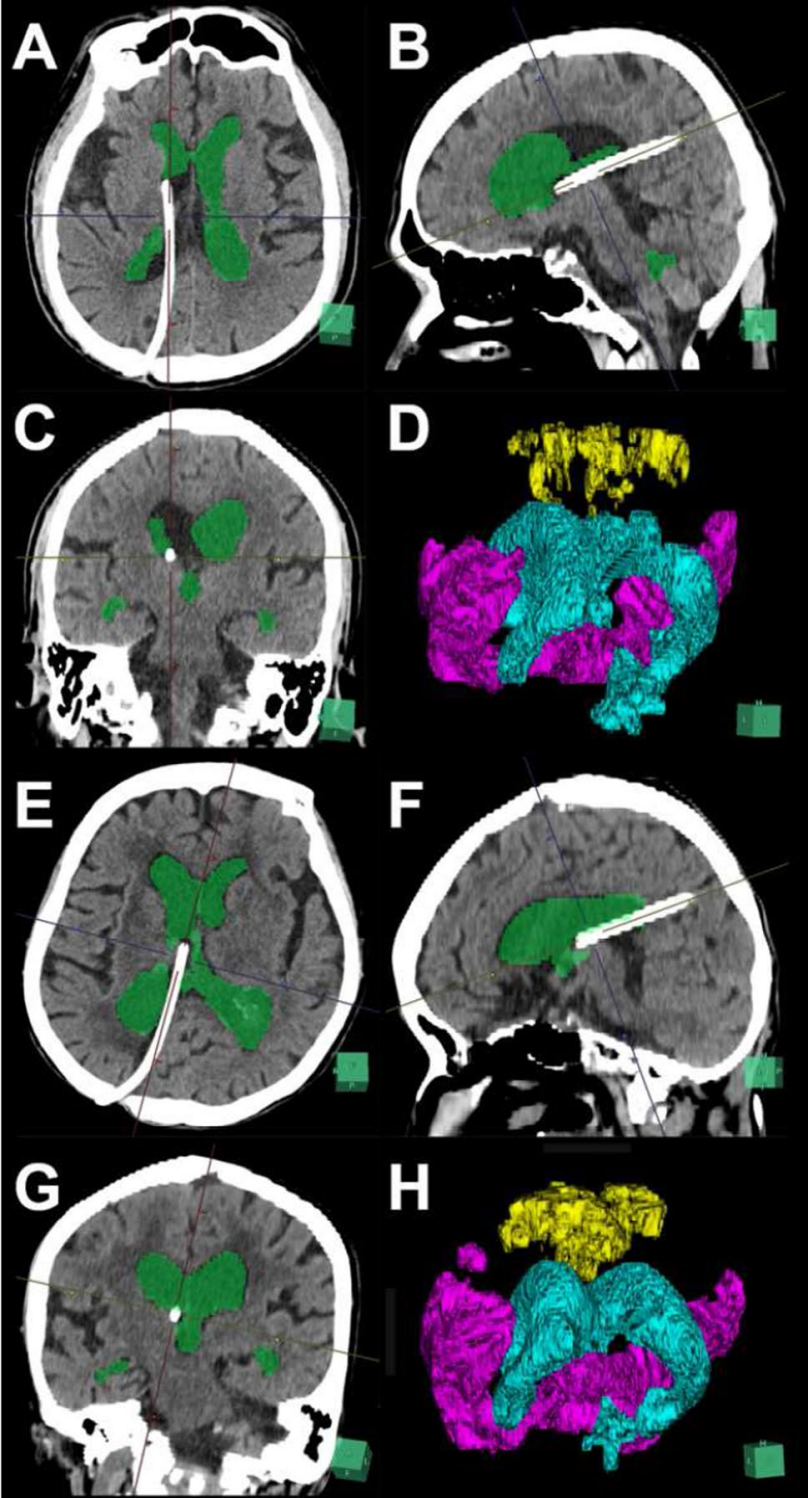
Performance of AI models from version 7 to version 9 on postoperative CT scans with 2.0-mm slice thickness. Segmentation masks were generated from postoperative plain CT images with a slice thickness of 2.0 mm in a 69-year-old male patient with iNPH, using AI models from Version 7 (**A–D**) to Version 9 (**E–H**). **A–C**, **E–G**: Total intracranial cerebrospinal fluid (CSF) space (green). **D**, **H**: Total ventricles (sky blue), high-convexity subarachnoid space (yellow), and Sylvian fissures with basal cisterns (magenta)

The model development was divided into two distinct phases: an Accuracy Optimization Phase (Versions 1–7), during which synthetic CT images were gradually replaced with manually labeled real CT data to enhance segmentation accuracy, and a Generalization Expansion Phase (Versions 8–11), where real CT images with varying slice thicknesses (Fig. 3) and postoperative features (Fig. 4) were added to broaden the model’s clinical applicability. In total, 172 real CT images (DESH 65, non-DESH 107) were used for training (53 and 89, respectively) and internal validation (12 and 18).

**Fig. 5:**
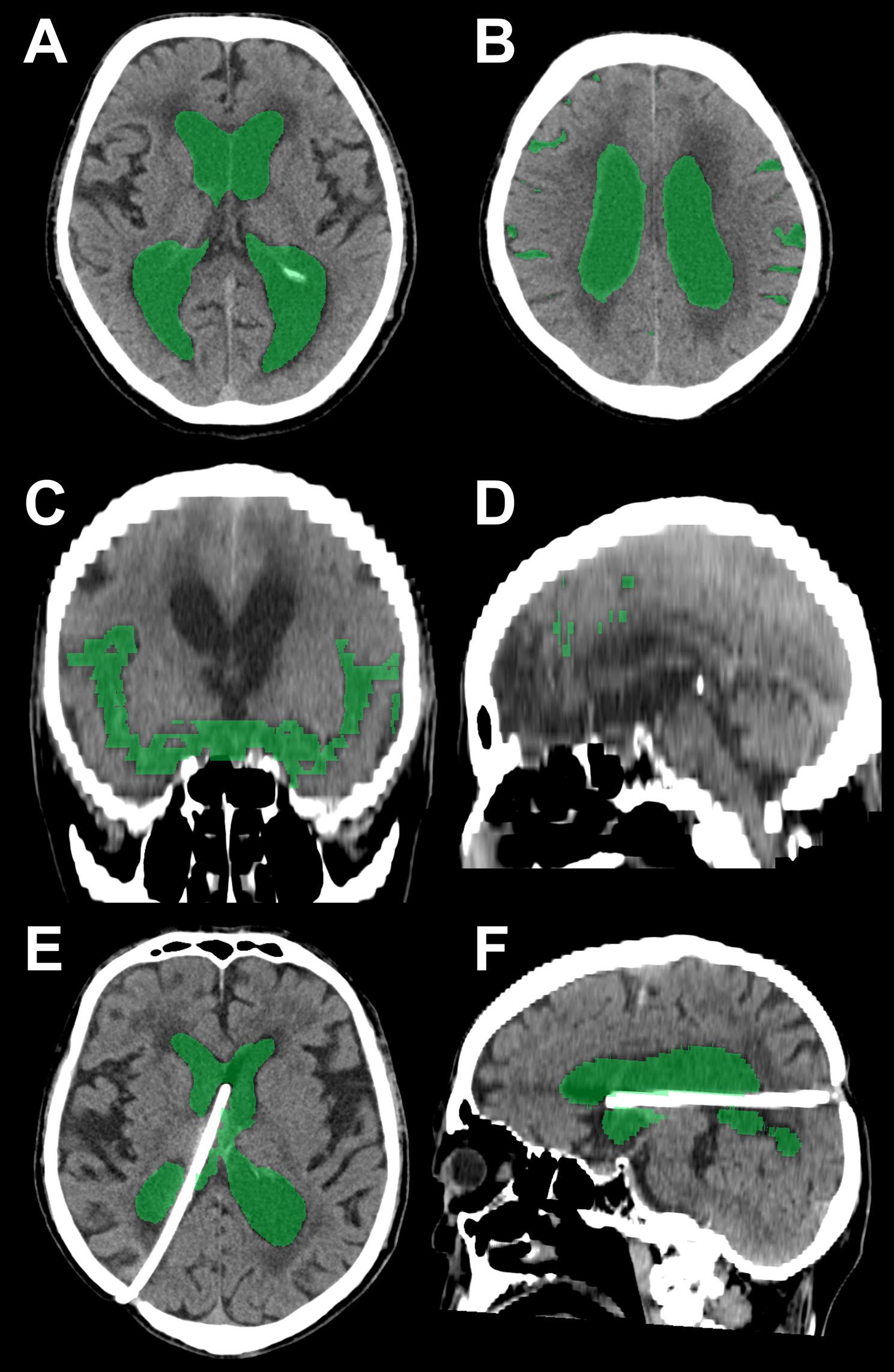
Region Extraction by Semantic Segmentation Using the Final AI Model (Version 10). **A**, **B**: Total ventricular regions (green) extracted from 5.0-mm plain CT images of a patient with iNPH. **C**: Sylvian fissures and basal cisterns (green) segmented from the same 5.0-mm CT scan. **D**: High-convexity subarachnoid space (green) segmented from the same 5.0-mm CT scan. **E, F**: Total ventricular region (green) extracted from a 2.0-mm postoperative CT scan of the same patient in axial (E) and sagittal (F) views.

Training and validation were repeated until the Dice score reached a plateau, enabling fully automatic segmentation of the target CSF regions from plain CT scans (Fig. 5). In internal validation, the Dice score for the total ventricles exceeded 0.9 and that for the total intracranial CSF space exceeded 0.8 at Version 7, with no further improvement through Version 11. Conversely, the score for the high-convexity SAS decreased from 0.53 in Version 10 to 0.47 in Version 11, and that for the Sylvian fissure and basal cistern decreased from 0.79 to 0.78.

**Fig. 6:**
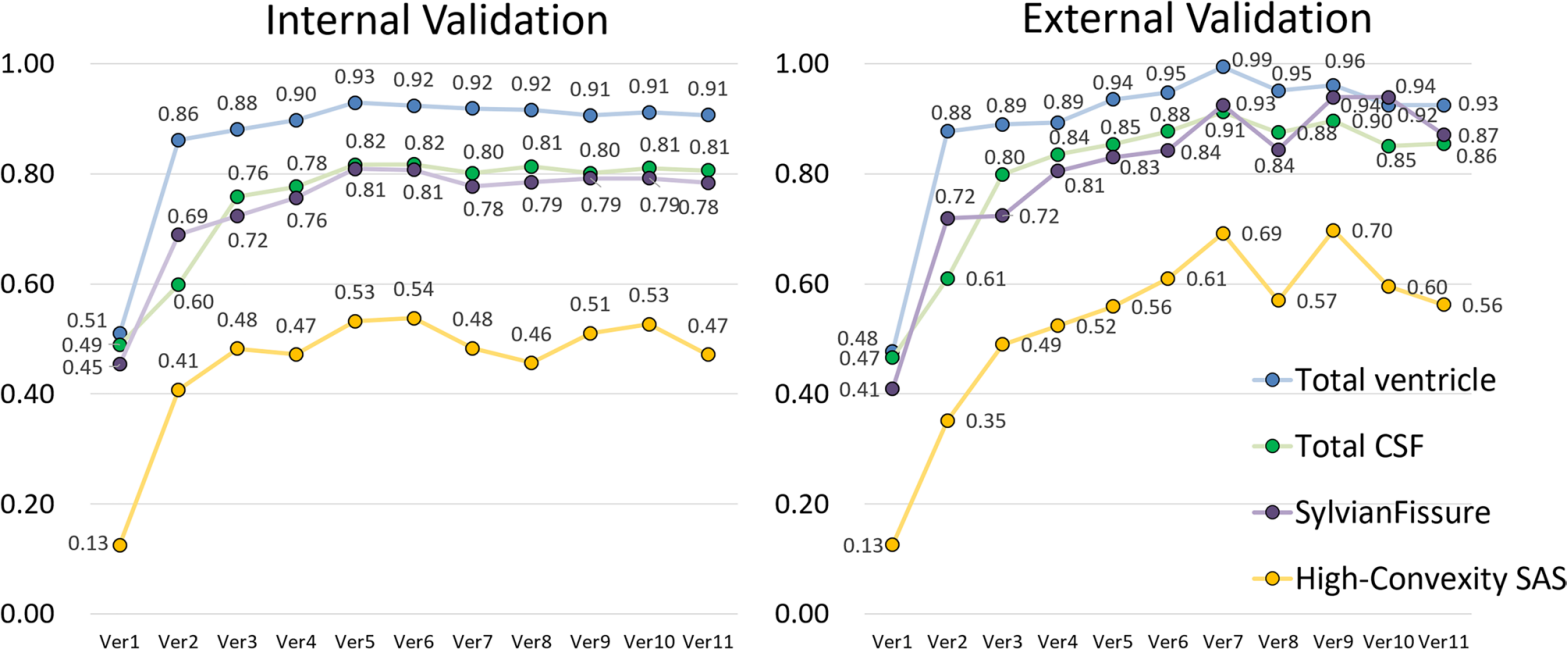
Changes in dice scores for four regions automatically extracted by semantic segmentation. Dice scores from internal validation (left) and external validation (right) for the total ventricles (blue), total intracranial CSF space (green), Sylvian fissures and basal cisterns (purple), and high-convexity subarachnoid space (SAS; yellow) in semantic segmentation models from Version 1 to Version 11, evaluated against ground truth masks

For external validation, 41 head CT images (slice thickness: 0.63–5 mm) from 30 patients diagnosed with DESH (11 males, 19 females; mean age, 84.2 ± 8.8 years) were used (Table 1). Among the 16 patients with iNPH and DESH who underwent VPS, both preoperative scans (slice thickness: 1–5 mm) and postoperative scans (slice thickness: 1–2 mm; acquired 1 day to 6 years after VPS) were available for 11 patients and included in the external validation. As controls, 30 head CT images (slice thickness: 0.63–2 mm) from 30 elderly patients diagnosed with non-DESH and brain atrophy (12 males, 18 females; mean age, 93.5 ± 3.4 years) were used for comparison (Table 1). Using all 71 real CT images prepared for external validation, the four target regions were automatically segmented with deep learning models from Version 7 through Version 11, and the corresponding Dice scores were calculated (Fig. 6).

**Table 1:** Mean ± SD (range) of segmented volumes and indices in the first validation set (*n* = 60; 30 DESH and 30 non-DESH)

	non-DESH (*n* = 30)	DESH (*n* = 30)		P1	P2
Male: Female	12: 18	11: 19			
Age (Mean ± SD)	90.9 ± 5.6	83.7 ± 8.8		0.001	
	(77–104)	(68–100)		0.001	
		Pre-Shunt	Post-Shunt		
Ventricle volume (mL)	66.9 ± 28.4	106.6 ± 35.2	104.5 ± 29.8	< 0.001	0.002
	(27.7–148.8)	(34.3–167.9)	(57.0–151.5)		
HCS volume (mL)	29.8 ± 12.7	3.9 ± 3.5	7.0 ± 3.8	< 0.001	0.023
	(5.9–53.6)	(0.3–12.9)	(0.7–12.6)		
Syl + BC volume (mL)	50.8 ± 9.8	62.1 ± 15.0	56.2 ± 17.7	0.002	0.003
	(31.0–70.5)	(33.3–89.1)	(22.3–83.8)		
CSF (mL)	310.5 ± 65.0	312.4 ± 67.8	318.7 ± 90.9	1.000	0.203
	(207.7–457.2)	(222.5–501.6)	(147.7–503.7)		
DESH index	5.1 ± 3.2	119.9 ± 149.6	46.0 ± 64.9	< 0.001	0.047
	(1.7–13.5)	(12.7–612.9)	(10.7–241.9)		
Venthi index	2.8 ± 1.7	76.2 ± 95.0	32.3 ± 50.9	< 0.002	0.049
	(0.7–6.9)	(6.4–375.2)	(7.4–188.7)		
Sylhi index	2.3 ± 1.7	43.7 ± 56.4	13.7 ± 14.7	< 0.003	0.046
	(0.9–8.8)	(5.4–237.7)	(3.3–53.2)		

*P*1: Probability values between the DESH (disproportionately enlarged subarachnoid space hydrocephalus) and non-DESH groups were calculated using the Mann–Whitney–Wilcoxon test

*P*2: Paired probability values between the pre-shunt surgery (Pre-Shunt) and post-shunt surgery (Post-Shunt) groups were calculated using the paired t-test

DESH index = (total ventricular volume + Sylvian fissure and basal cistern volume) / high-convexity subarachnoid space volume

Venthi index = total ventricular volume / high-convexity subarachnoid space volume

Sylhi index = Sylvian fissure and basal cistern volume / high-convexity subarachnoid space volume

**Fig. 7:**
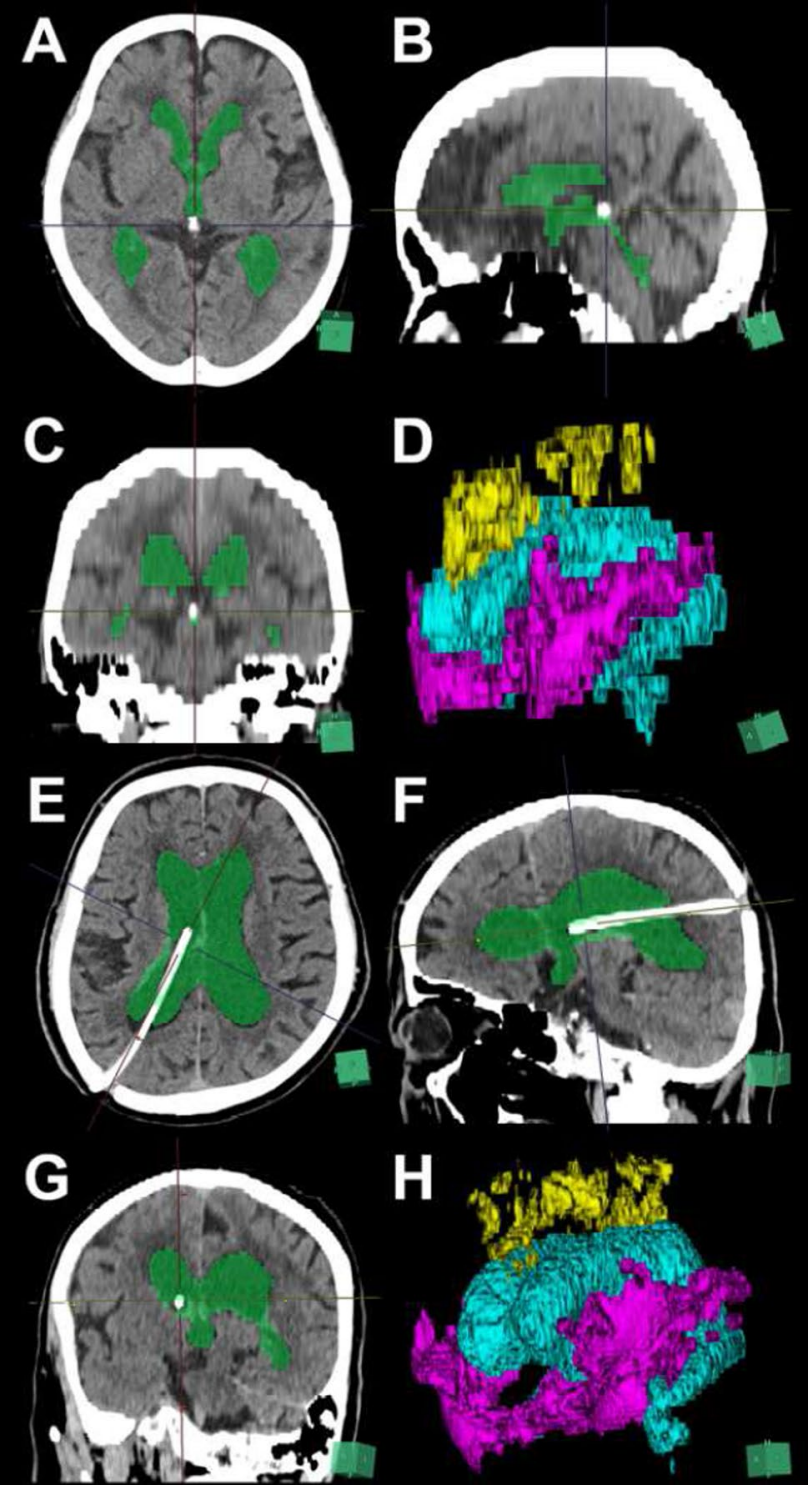
Semantic segmentation results using the final model (version 10). **A–D**: Segmentation masks generated from plain CT images with a slice thickness of 5.0 mm in an 81-year-old female patient with iNPH. **E–H**: Segmentation masks generated from postoperative plain CT images with a slice thickness of 2.0 mm in a 73-year-old female patient with iNPH. **A–C**, **E–G**: Total ventricles (green). **D**, **H**: Total ventricles (sky blue), high-convexity subarachnoid space (yellow), and Sylvian fissures with basal cisterns (magenta)

The Dice score for the total ventricles increased slightly from 0.92 in Version 10 to 0.93 in Version 11, and that for the total intracranial CSF space increased from 0.85 to 0.86. In contrast, the score for the high-convexity SAS decreased from 0.60 in Version 10 to 0.56 in Version 11, and that for the Sylvian fissure and basal cistern decreased from 0.94 to 0.87. Based on these results, Version 10 was adopted as the final model (Fig. 7).

### Regional volumes and indices obtained with the final model

Using the final Version 10 semantic segmentation model, VOIs were automatically extracted from plain head CT images of 30 patients with DESH and 30 patients with non-DESH (brain atrophy) included in the external validation. The results are summarized in Table 1. Compared with the non-DESH group, the DESH group had significantly larger total ventricular volume and Sylvian fissure and basal cistern volume, and significantly smaller high-convexity SAS volume, while no significant difference was observed in total CSF volume. All three indices, DESH index, Venthi index, and Sylhi index, were markedly higher in the DESH group. In 11 patients with iNPH who had both pre- and postoperative CT scans available, postoperative values were compared with preoperative values. Postoperatively, total ventricular volume and Sylvian fissure and basal cistern volume were significantly reduced, while high-convexity SAS volume was significantly increased. All three indices showed significant postoperative reductions, whereas the decrease in total CSF volume did not reach statistical significance.

### Diagnostic performance and optimal cut-off values for DESH-related indices

**Fig. 8:**
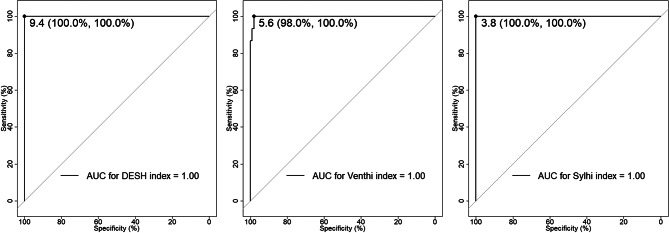
Receiver operating characteristic (ROC) curves for DESH detection. Candidate optimal cutoff points (with corresponding sensitivity and specificity values) are indicated by black markers. DESH index = (Total ventricle volume) + (Sylvian fissure and basal cistern volume) / (High-convexity part of the subarachnoid space volume). Venthi index = (Total ventricle volume) / (High-convexity part of the subarachnoid space volume). Sylhi index = (Sylvian fissure and basal cistern volume) / (High-convexity part of the subarachnoid space volume)

In the second validation set, consisting of 115 consecutive patients who underwent plain head CT over a two-month period (56 males, 59 females; mean age, 77.5 ± 13.3 years), 15 patients (13%) were classified as having DESH. DESH was observed only in patients aged ≥ 68 years. Among the 97 patients aged ≥ 65 years, the prevalence of DESH was 15.5%. The distributions of age, sex, and the four volumetric parameters and three derived indices obtained by AI-based segmentation are shown in Table 2. These distributions were comparable to those observed in the first validation set (30 DESH vs. 30 non-DESH). The AUC of the DESH index for detecting DESH was 1.00, with a sensitivity of 100% and a specificity of 100% at the optimal cutoff value of 9.4 (Fig. 8). The Venthi and Sylhi indices also showed perfect diagnostic performance, with AUCs of 1.00 at optimal cutoff values of 5.6 and 3.8, respectively.

**Table 2:** Mean ± SD (range) of segmented volumes and indices in the second validation set (*n* = 115)

	non-DESH (*n* = 100)	DESH (*n* = 15)	*P*
Male: Female	46: 54	10: 5	
Age (Mean ± SD)	77.4 ± 14.0	78.3 ± 7.1	0.143
	(27–97)	(68–88)	
Ventricle volume (mL)	53.3 ± 23.7	93.2 ± 18.8	< 0.001
	(8.9–140.3)	(70–131)	
HCS volume (mL)	27.0 ± 10.1	7.6 ± 4.6	< 0.001
	(8.3–56.4)	(0.4–13.4)	
Syl + BC volume (mL)	39.7 ± 15.2	54.6 ± 14.9	0.002
	(9.8–76.1)	(31.4–87.8)	
CSF (mL)	252 ± 82.4	276 ± 61.2	0.3
	(55.6–448.7)	(164–384)	
DESH index	3.8 ± 1.8	71.0 ± 106.0	< 0.001
	(0.8–8.3)	(10.5–355.0)	
Venthi index	2.2 ± 1.2	45.5 ± 68.1	< 0.001
	(0.3–6.4)	(5.8–230.3)	
Sylhi index	1.6 ± 0.7	25.5 ± 38.0	< 0.001
	(0.5–3.8)	(3.9–124.7)	

When integer cutoff values were applied, with thresholds of 10, 6, and 4 for the DESH, Venthi, and Sylhi indices, respectively, the corresponding AUCs, sensitivities, and specificities are presented in Table 3. The AUC of the DESH index remained unchanged at a cutoff value of 10.

**Table 3:** Diagnostic performance of the Deep-learning algorithm for detecting disproportionately enlarged subarachnoid space hydrocephalus (DESH) on CT

	Cut-off	DESH (15)	non-DESH (100)	AUC	Sensitivity	Specificity
		upper/ lower	upper/ lower		%	%
DESH index	10	15/0	0/100	1.00	100	100
Venthi index	6	14/1	2/98	0.96	93.3	98.0
Sylhi index	4	14/1	0/100	0.97	93.3	100

DESH index = (total ventricular volume + Sylvian fissure and basal cistern volume) / high-convexity subarachnoid space volume

Venthi index = total ventricular volume / high-convexity subarachnoid space volume

Sylhi index = Sylvian fissure and basal cistern volume / high-convexity subarachnoid space volume

AUC: area under the receiver-operating characteristic curve

Sens, sensitivity; spec, specificity

## Discussion

In this study, we developed an automatic segmentation of intracranial CSF space and three VOIs associated with DESH from plan head CT scan with 0.45–5.0 mm thickness. Plain CT imaging, with its lower spatial resolution, such as 5.0 mm slice intervals, presents greater challenges for the automatic segmentation of brain regions, ventricles, and CSF spaces compared to the higher resolution of 3-Tesla 3D MRI. However, the application of advanced deep learning approaches has made it possible to detect these VOIs from plain CT images. Although ventricular enlargement is the most common and reliable imaging marker for hydrocephalus, the concurrent expansion of the Sylvian fissures in iNPH often leads to misdiagnosis as brain atrophy. This underscores the limitation of relying solely on automated ventricular segmentation. If only the ventricles are segmented and ventricular enlargement alone is assessed, cases of iNPH may be missed, necessitating DESH evaluation in the diagnostic workflow. However, the automatic segmentation of complex structures such as the high-convexity SAS, Sylvian fissures and basal cisterns has remained challenging, even with 3D MRI, and no prior studies have addressed this issue. In our previous work, we developed a deep learning model capable of automatically extracting the ventricles, high-convexity SAS, Sylvian fissures and basal cisterns from 3D MRI data [21]. In the current study, cycle-GAN was used to convert MRI data with segmentation annotations into synthetic CT images (Fig. 1 and Additional file 1), which were subsequently used to train a 3D U-Net model for semantic segmentation. However, in the initial model (Version 1), which was trained solely on synthetic CT images, satisfactory segmentation was not achieved, as reflected by considerably low Dice scores (Fig. 6). By incrementally refining the training dataset, replacing 20% of synthetic CT images with real CT images in each cycle, we ultimately developed a 3D U-Net model trained exclusively on real plain head CT data. In Version 2, in which only 17 synthetic CT images were replaced with real CT images, the Dice scores for all four regions improved dramatically, and they continued to increase until Version 7, when all synthetic CT images had been replaced with real CT images. Although reducing slice thickness in CT images generally improves spatial resolution by minimizing the partial volume effect, it also increases image noise, which impeded model training. In fact, noise levels increased by more than threefold when the slice thickness was reduced from 5 mm to 0.5 mm. For brain CT imaging, a slice thickness of 2.5 to 5 mm has been reported to provide an optimal balance between spatial resolution and image noise [24].

This model represents a novel achievement in the automated extraction of both the high-convexity SAS and the Sylvian fissures with basal cisterns from plain CT images, with no comparable results reported by other research groups to date. The AI-based segmentation appeared to outperform manual segmentation performed by experts. Although DESH has recently gained international recognition for its contribution to improving diagnostic accuracy in iNPH, its evaluation remains subjective and susceptible to inter-observer variability [17–20], underscoring the need for quantitative, objective indicators. The present AI model addresses these limitations by providing fully automated, quantitative, and reproducible measurements, enabling objective assessment of DESH in both clinical and research settings. With this novel AI-CAD application, iNPH may be assessed more objectively by providing quantitative imaging biomarkers, which can serve as supportive evidence alongside clinical evaluation and other diagnostic procedures. As the condition is increasingly observed in older adults, including those over 80 years of age and sometimes with comorbidities such as stroke, the ability to quantify both DESH-related indices and symptom severity enables clinicians to tailor therapeutic strategies to individual patient profiles. We aim to promote the widespread adoption of this AI-CAD application to enhance the diagnosis and management of iNPH in clinical practice. Furthermore, quantitative assessment of changes in the DESH index and volumetric parameters on CT scans before and after VPS may provide valuable clinical insights. For example, even if ventricular size remains unchanged, a reduction in the volume of the Sylvian fissures and basal cisterns, accompanied by an increase in the volume of the high-convexity SAS and a decrease in the DESH and Sylhi indices, may indicate effective CSF drainage and rule out shunt malfunction. Conversely, persistent symptoms with an unchanged DESH index may suggest underdrainage, warranting shunt valve pressure adjustment, such as lowering the pressure setting.

This study has several limitations. First, domain shift, which refers to differences in imaging protocols and quality among facilities that can lower performance, is a common but critical issue in AI-based segmentation and detection [25]. Second, the reliability and validity of the VOIs automatically extracted from CT scans by these applications have not yet been fully verified in this study; further validation using VOIs derived from 3D MRI in the same patients is warranted. Finally, we did not investigate the relationship between the DESH index and the various symptoms of iNPH. Future studies should examine the association between the DESH index and symptom severity, as well as its impact on symptom improvement following shunt surgery. The rapid advancement of AI-CAD systems has revolutionized the field of automated medical image segmentation, offering new possibilities for objective and reproducible evaluation in clinical practice.

## Conclusions

In this study, our deep learning model achieved fully automated segmentation of the VOIs required for detecting DESH from plain head CT images. This AI-powered application enables quantitative evaluation of both the presence and severity of DESH, a key radiological marker for iNPH, thereby reducing the subjectivity and inter-observer variability of conventional assessments. Beyond diagnosis, it has the potential to support objective monitoring of treatment response after shunt surgery, including the identification of effective CSF drainage or underdrainage based on changes in DESH-related indices. By operating on widely available non-contrast CT, this tool may broaden access to timely and appropriate treatment, particularly for elderly patients and in clinical settings where MRI is not readily available.

## Supplementary Information

Below is the link to the electronic supplementary material.

Supplementary Material 1

Supplementary Material 2

Supplementary Material 3

## Data Availability

The CT data in this study is not available to the community via any open repositories, because the ethics committees have approved the sharing of the MRI data in this research with collaborative institutes and does not allow its being provided to other institutions. The data will be available only on the condition that the ethics committees approve any new participation in the collaborative research. Sharing the algorithm codes for these two applications is not possible, as they are commercialized as medical devices by FUJIFILM Corporation.

## Declarations

### Authors' information

SY began working as a neurosurgeon in 1997 and commenced his research at Kyoto University Graduate School of Medicine in 2001. Over the course of four years, SY acquired a knowledge in medical statistics and genetics. In 2017, SY initiated collaborations with Fujifilm Corporation on 3D/4D image analysis research.

## Abbreviations

AI-CAD: Artificial intelligence-based computer-aided diagnosis
AUC: Area under the receiver-operating characteristic curve
CSF: Cerebrospinal fluid
CT: Computed tomography
DESH: Disproportionately enlarged subarachnoid space hydrocephalus
GAN: Generative adversarial networks
iNPH: Idiopathic normal pressure hydrocephalus
MPRAGE: Magnetization-prepared rapid gradient echo
ROC: Receiver-operating characteristic curve
SAS: Subarachnoid spaces
SFD: Sylvian fissure dilation
THC: Tightened sulci in the high convexities
VOI: Volume of interest
VPS: Ventriculo-peritoneal shunt surgery
3D: Three-dimensional
4D: Four-dimensional
