# Supplementary material for: CT-based automatic segmentation of key CSF regions for detecting disproportionately enlarged subarachnoid space hydrocephalus
Source: Fluids Barriers CNS. 2026 Jun 23;23:83. doi: 10.1186/s12987-026-00814-5 (PMC13289119; doi:10.1186/s12987-026-00814-5)
Supplement: Supplementary file 1 — Supplementary Material 1 [file 12987_2026_814_MOESM1_ESM.pdf]

3D T1-weighted MRI

Synthetic CT Image

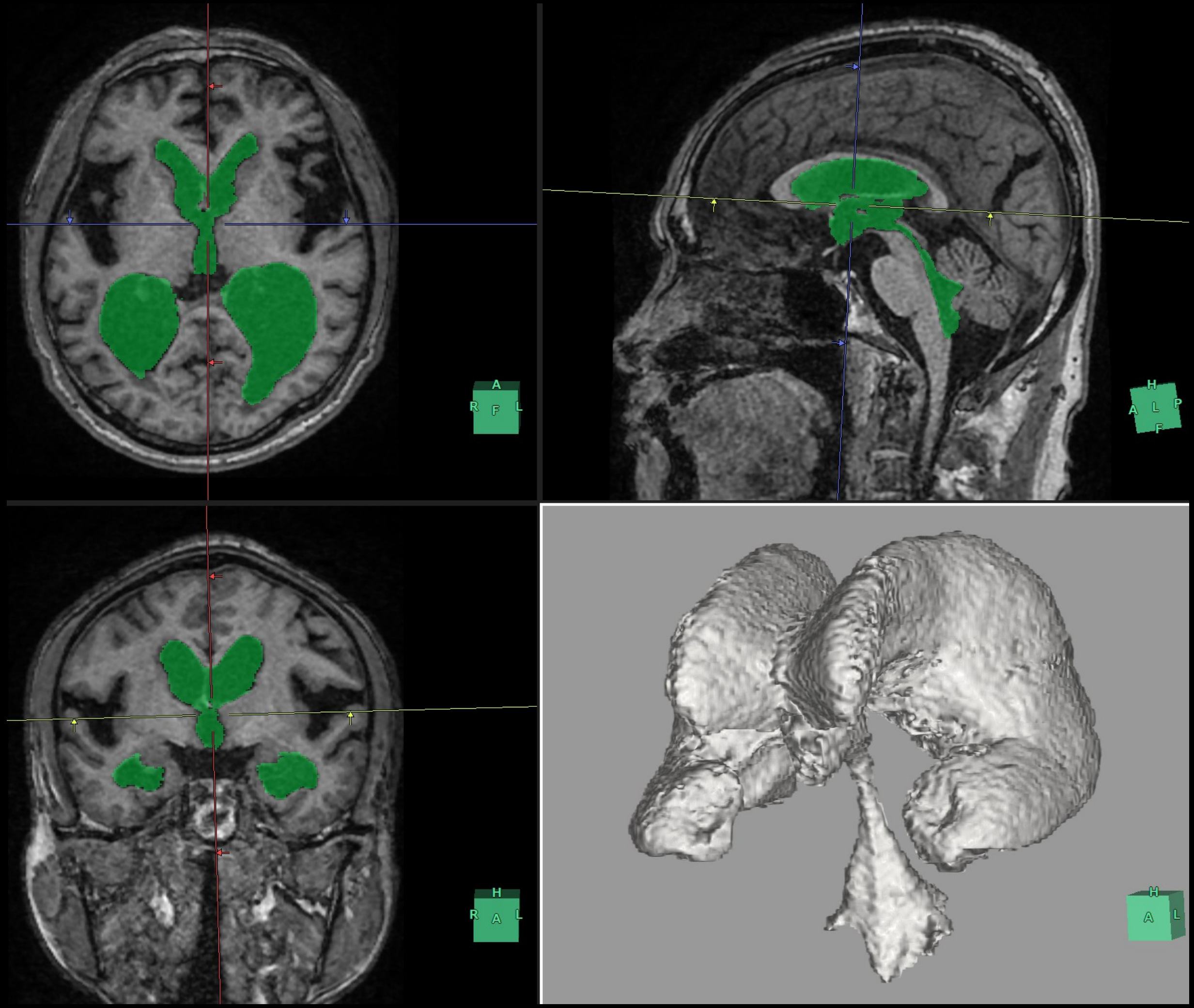

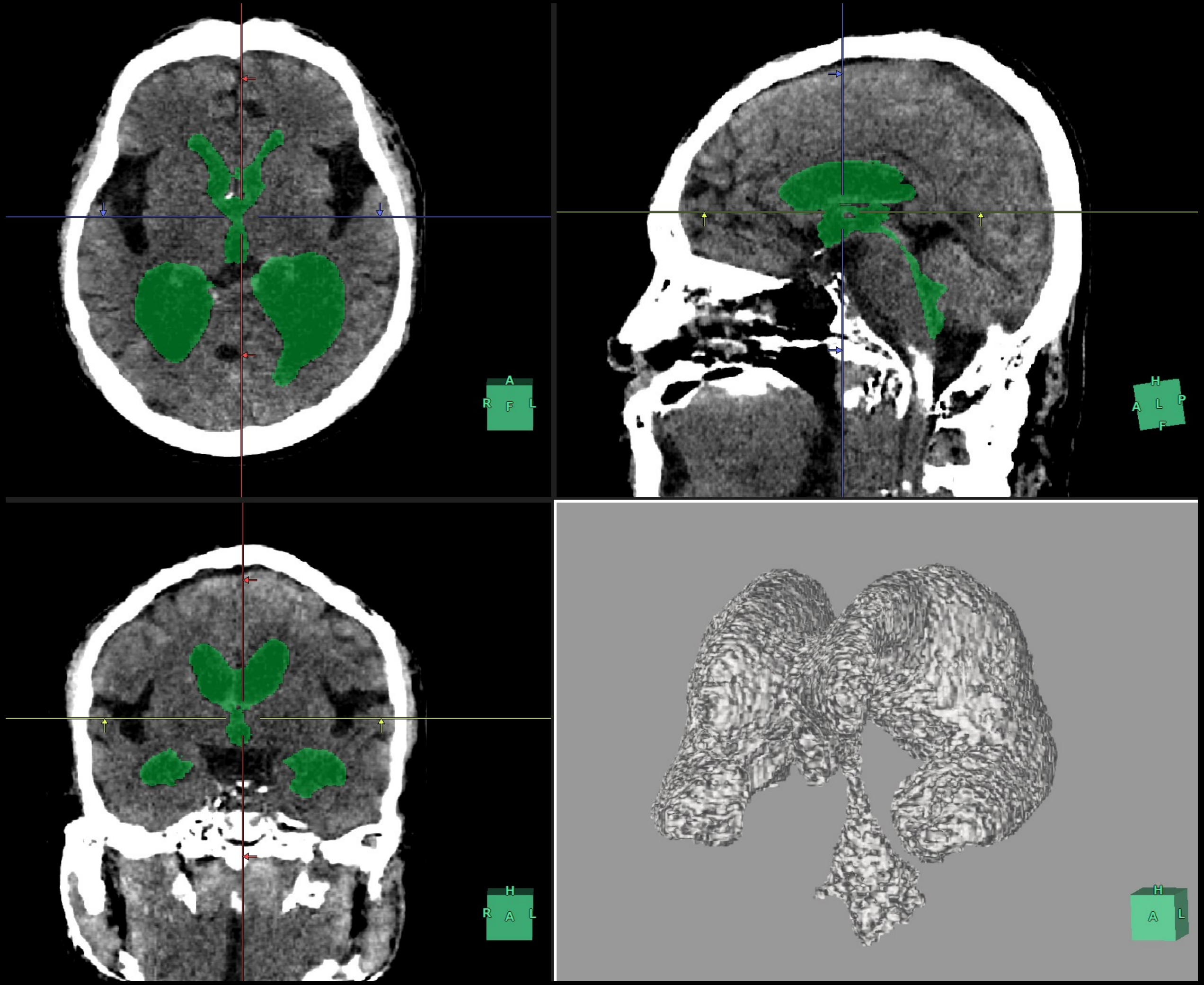

Hakim's disease (iNPH, 65y, Male) Total ventricle

3D T1-weighted MRI

Synthetic CT Image

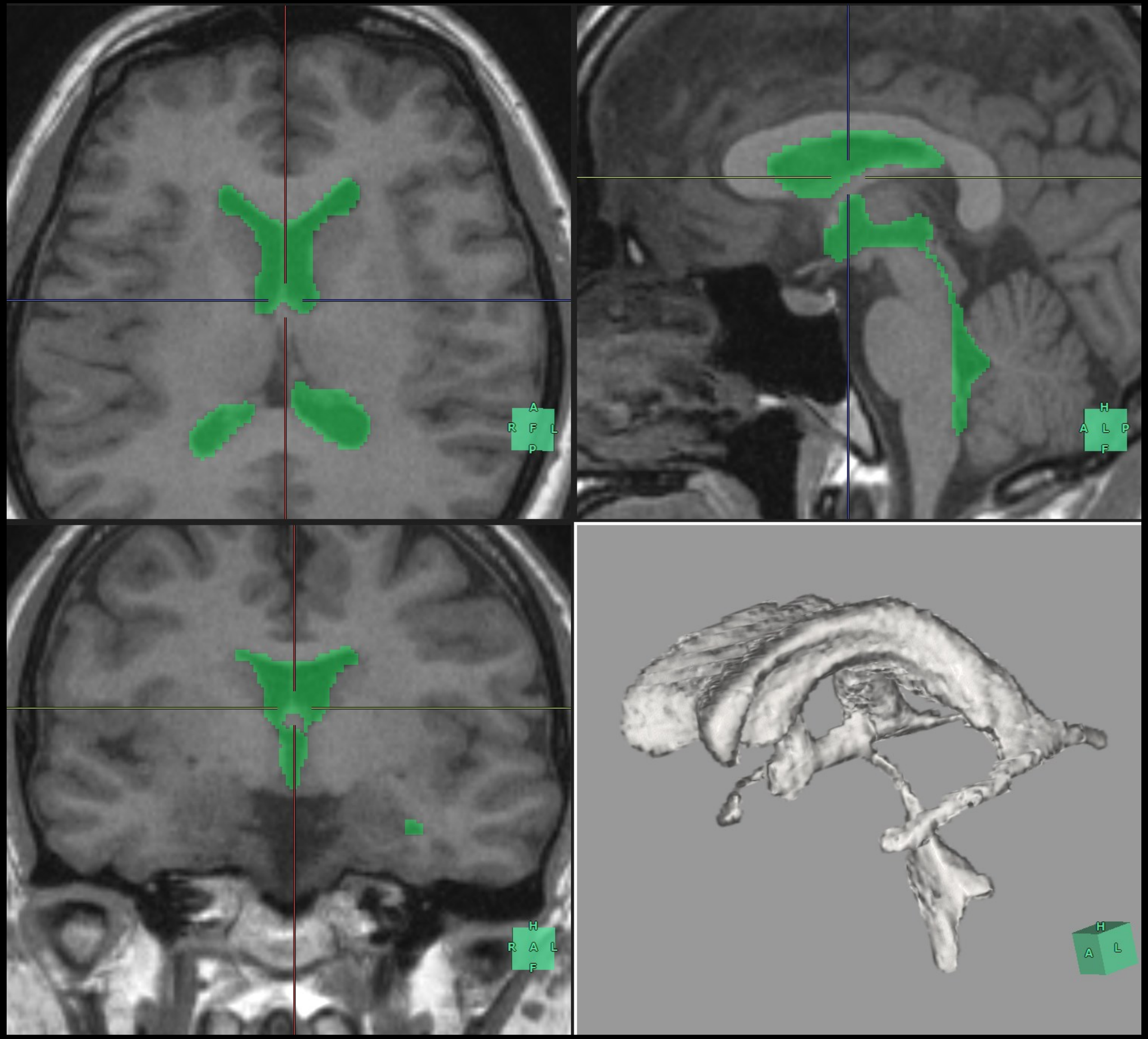

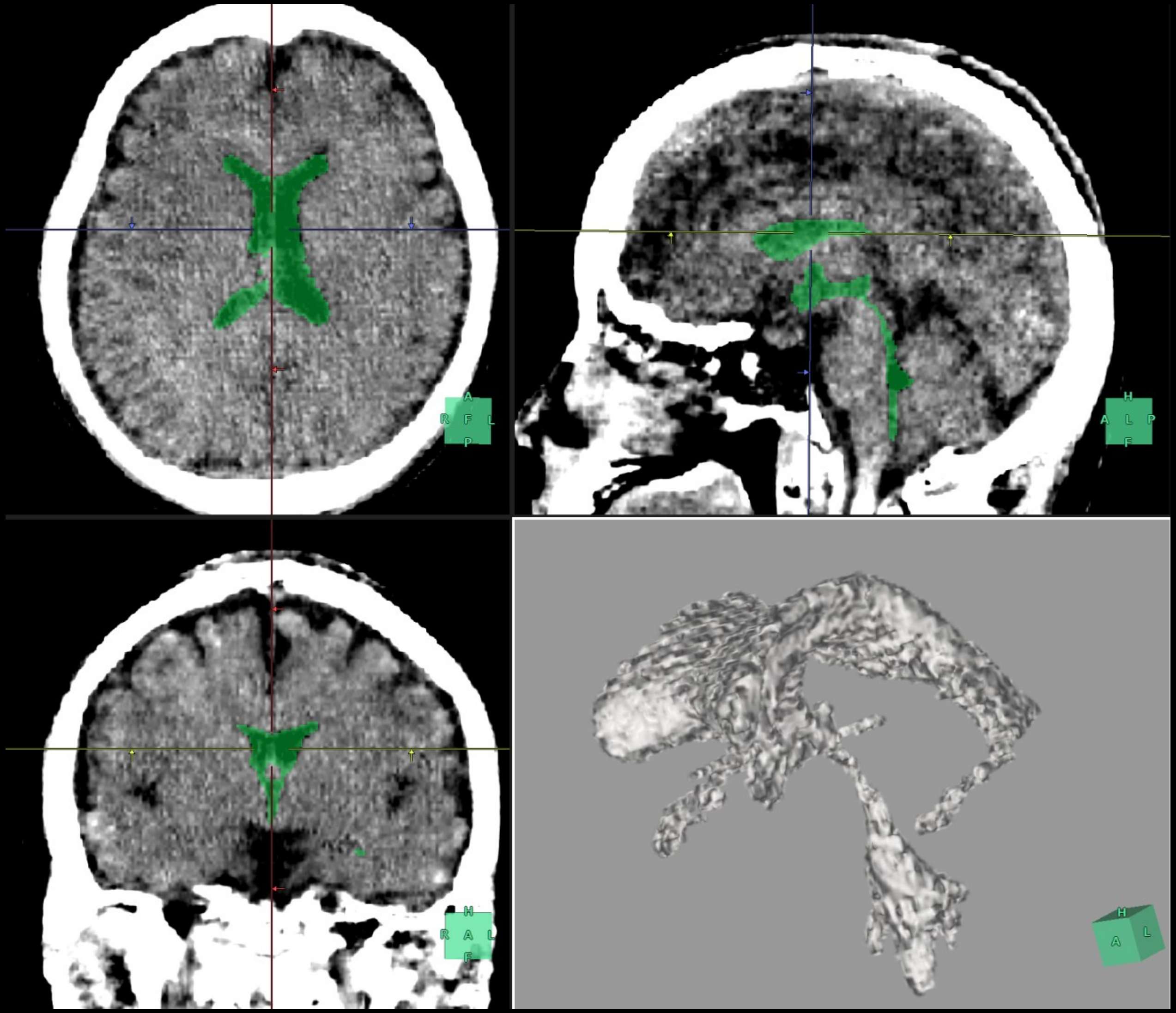

Normal (43y, Female) Total ventricle

3D T1-weighted MRI ⟳ Synthetic CT Image

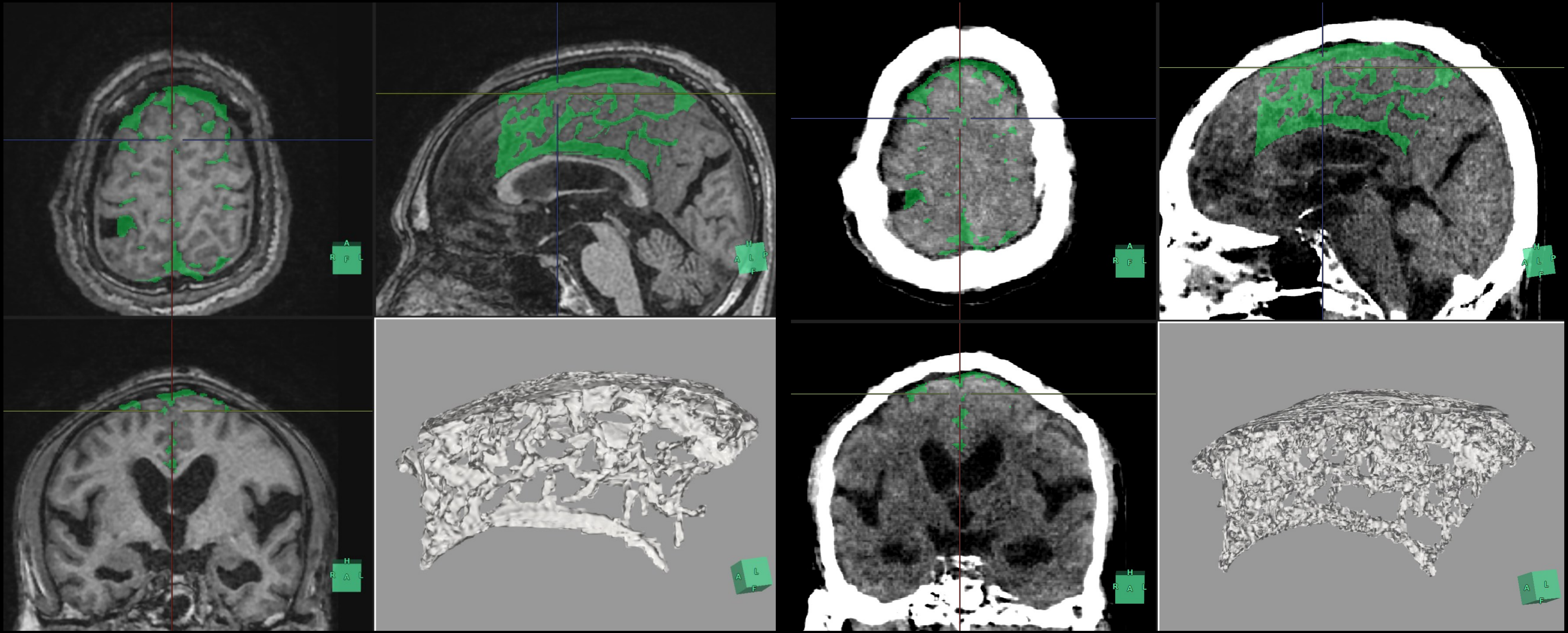

Hakim's disease (iNPH, 65y, Male) High-convexity subarachnoid space

3D T1-weighted MRI

Synthetic CT Image

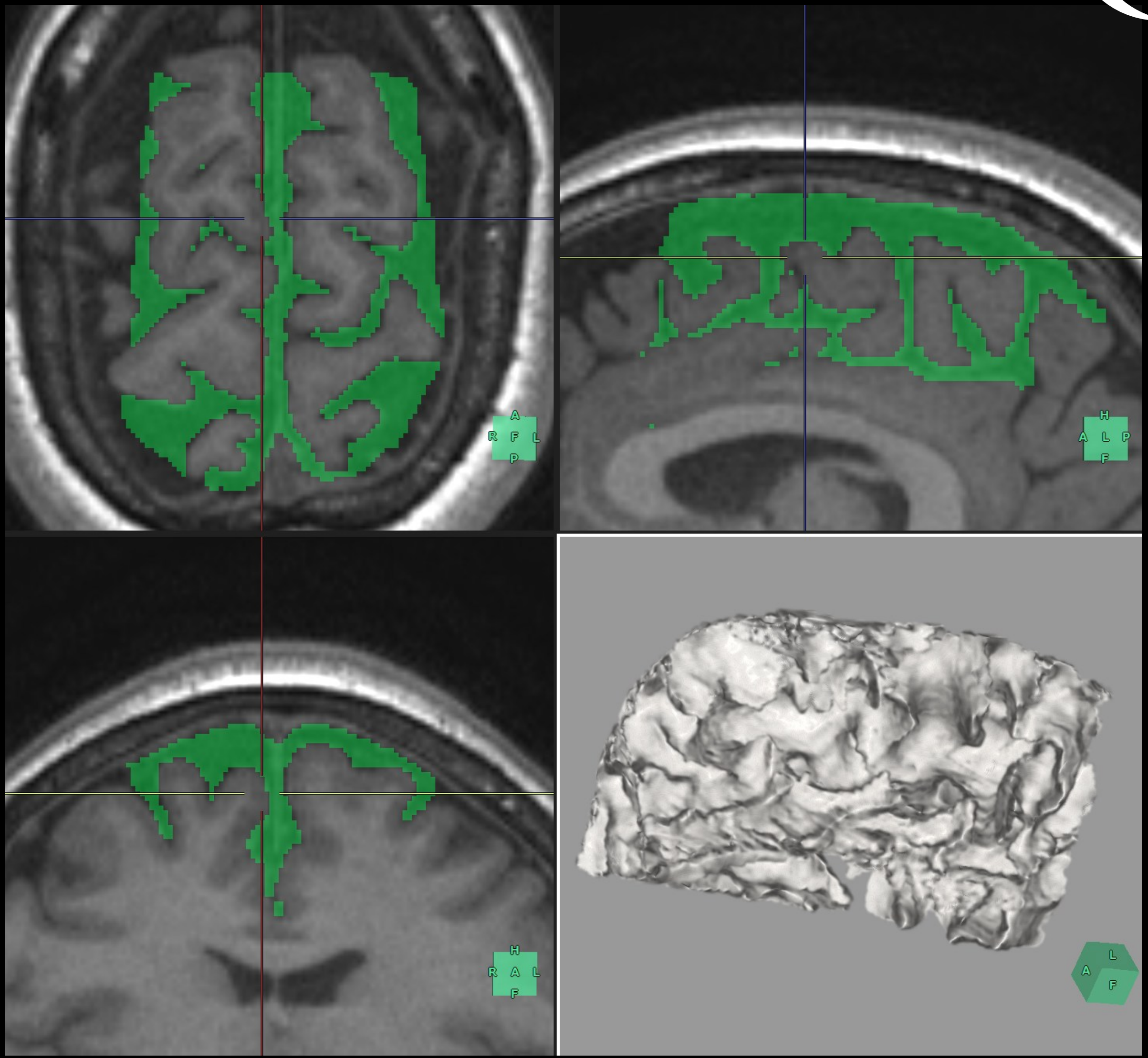

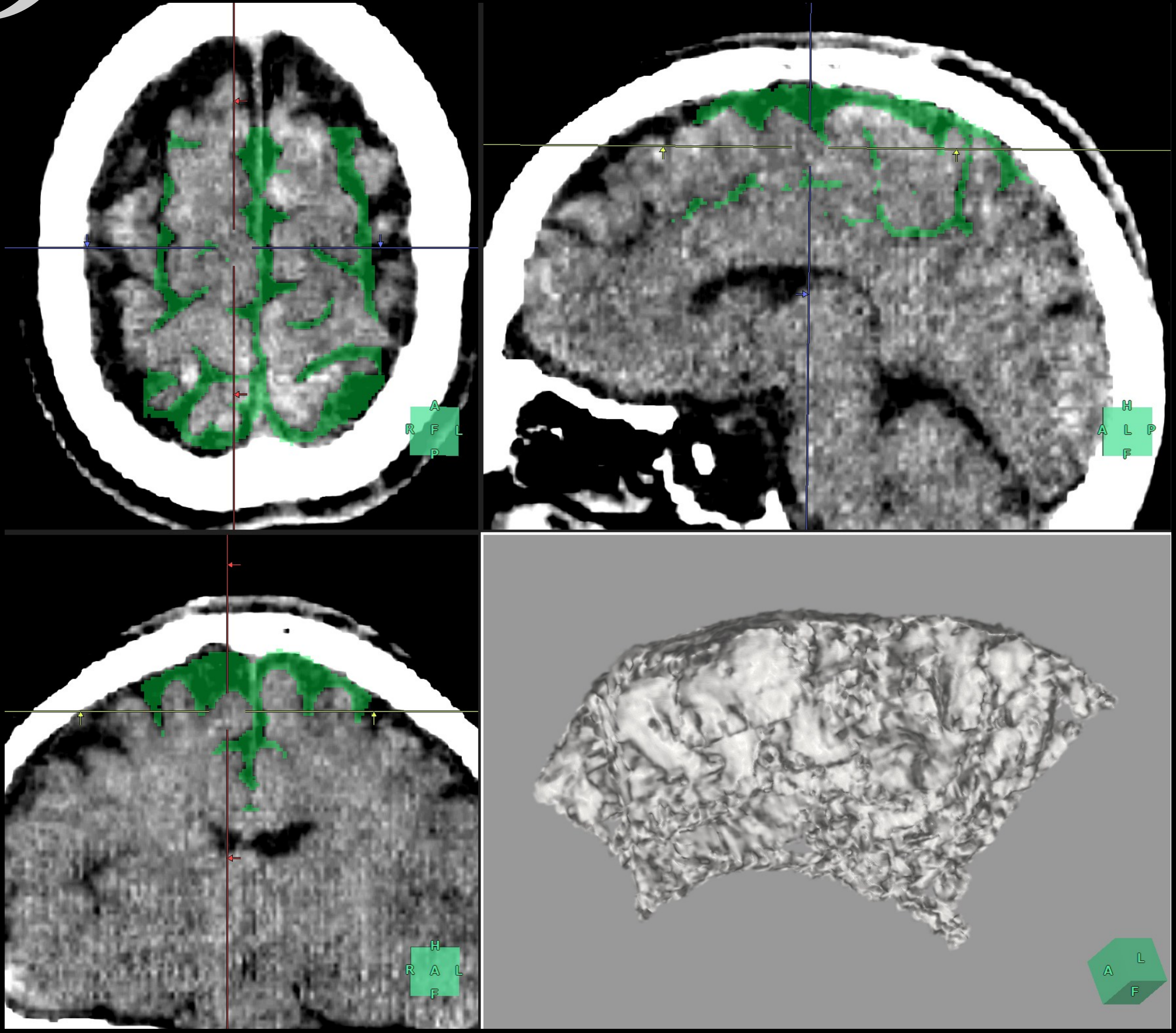

Normal (43y, Female) High-convexity subarachnoid space

3D T1-weighted MRI

Synthetic CT Image

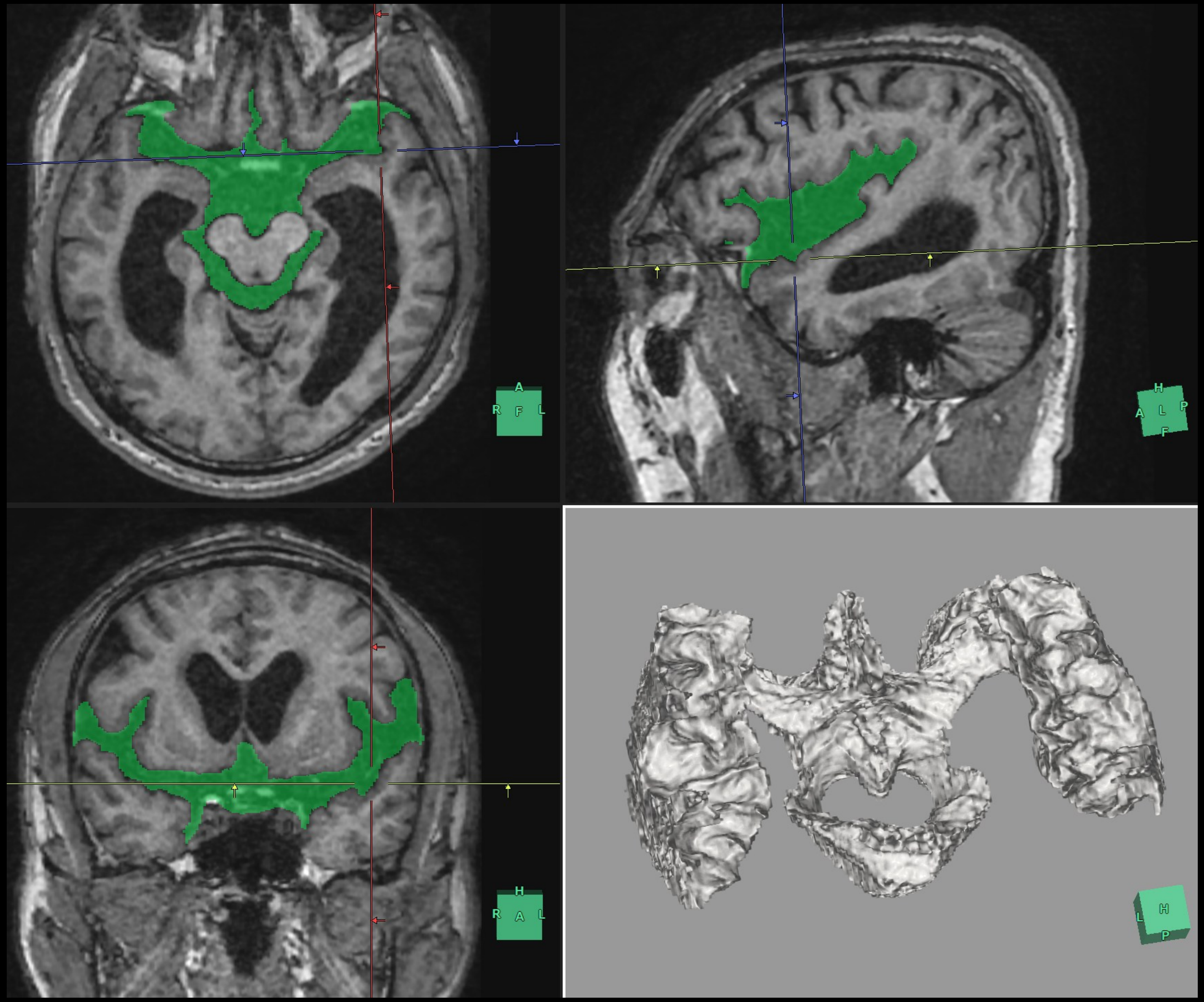

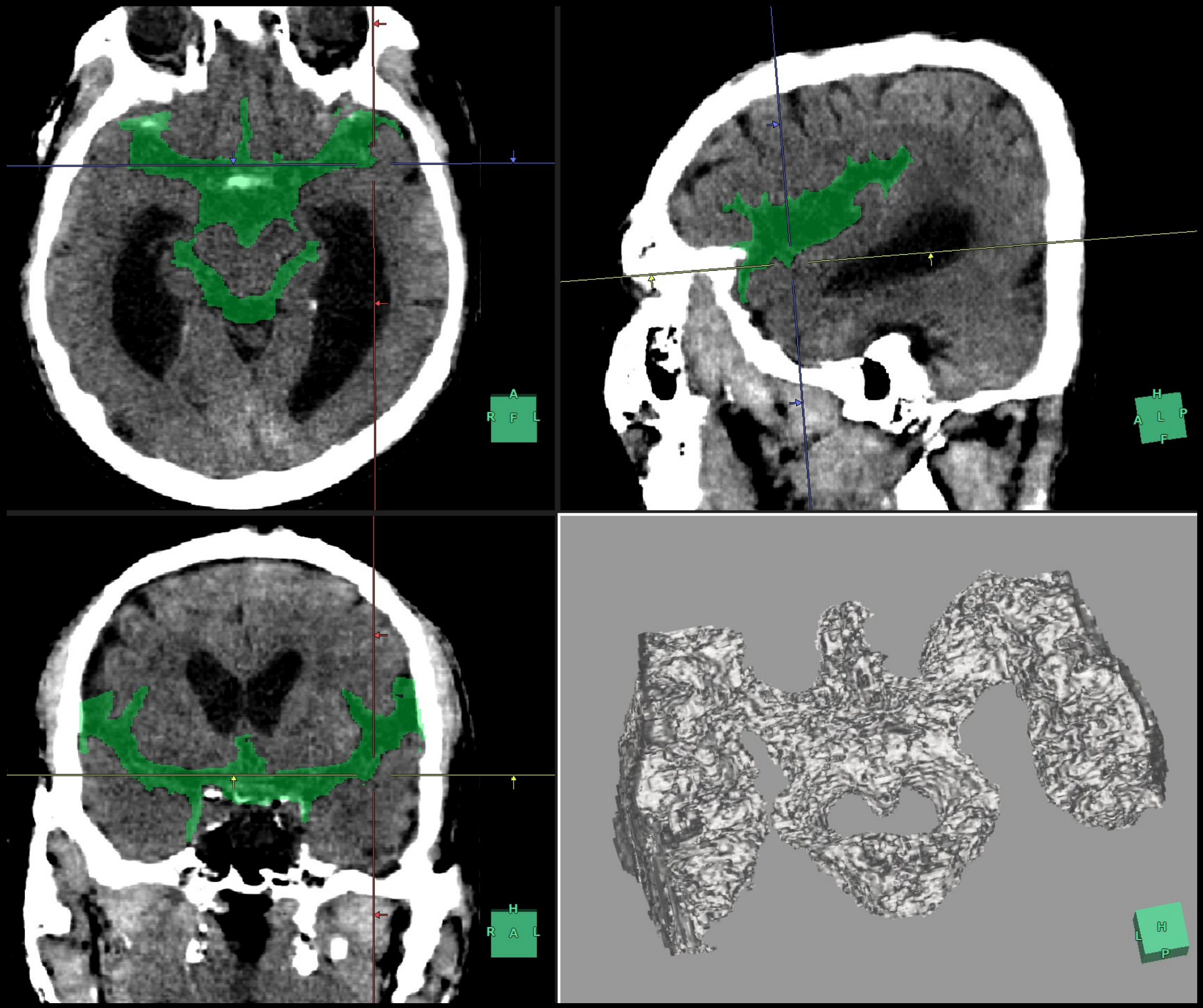

Hakim's disease (iNPH, 65y, Male) Sylvian fissure and basal cistern

3D T1-weighted MRI

Synthetic CT Image

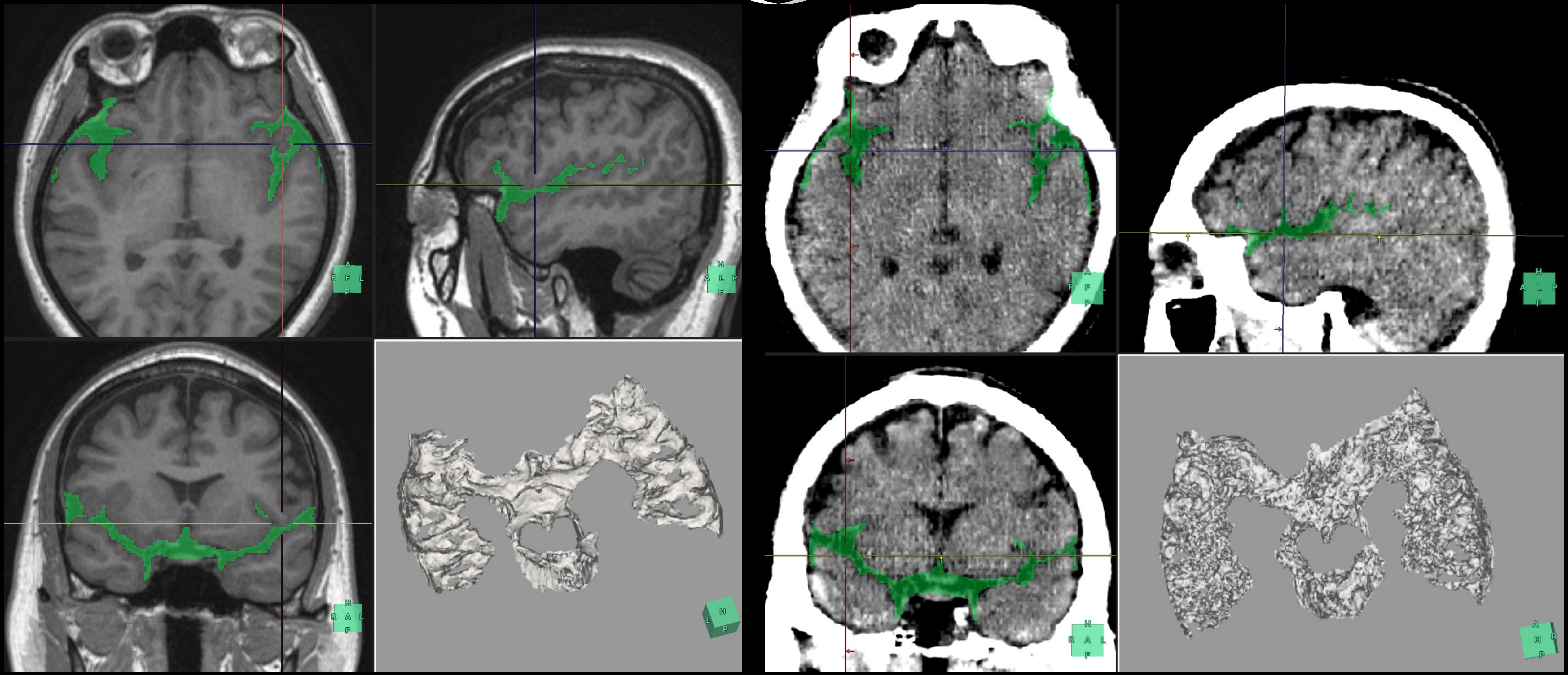

Normal (43y, Female) Sylvian fissure and basal cistern

3D T1-weighted MRI

Synthetic CT Image

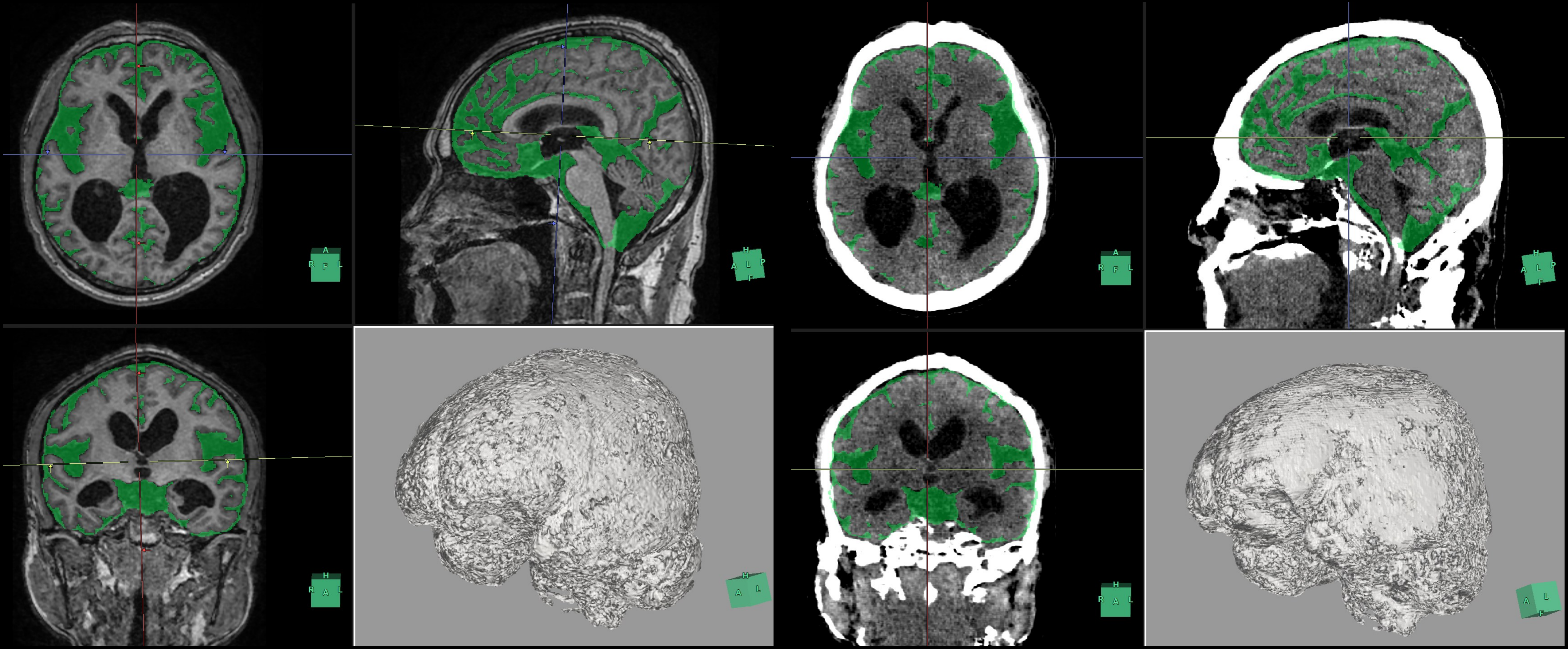

Hakim’s disease (iNPH, 65y, Male) Total subarachnoid space

3D T1-weighted MRI

Synthetic CT Image

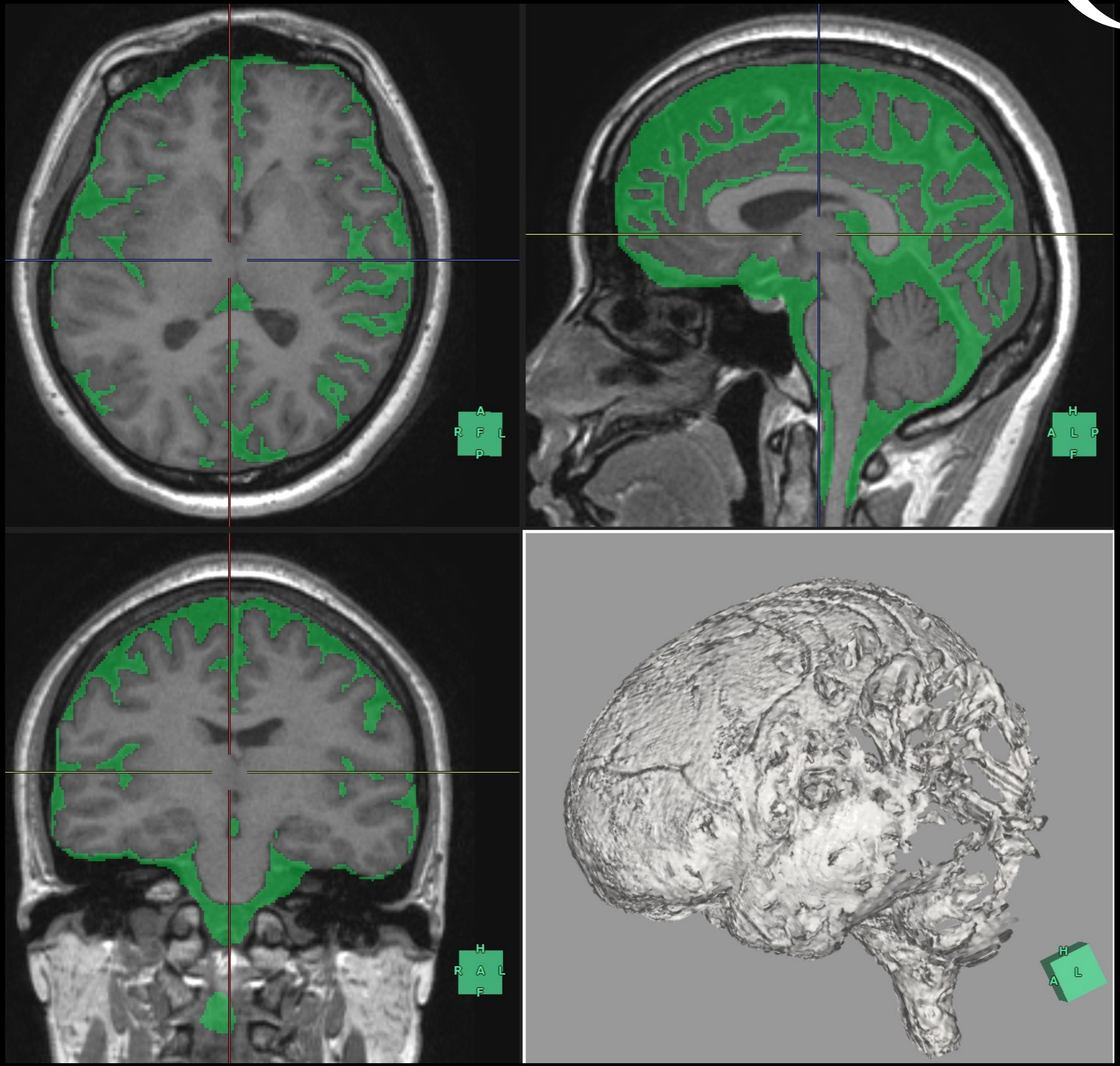

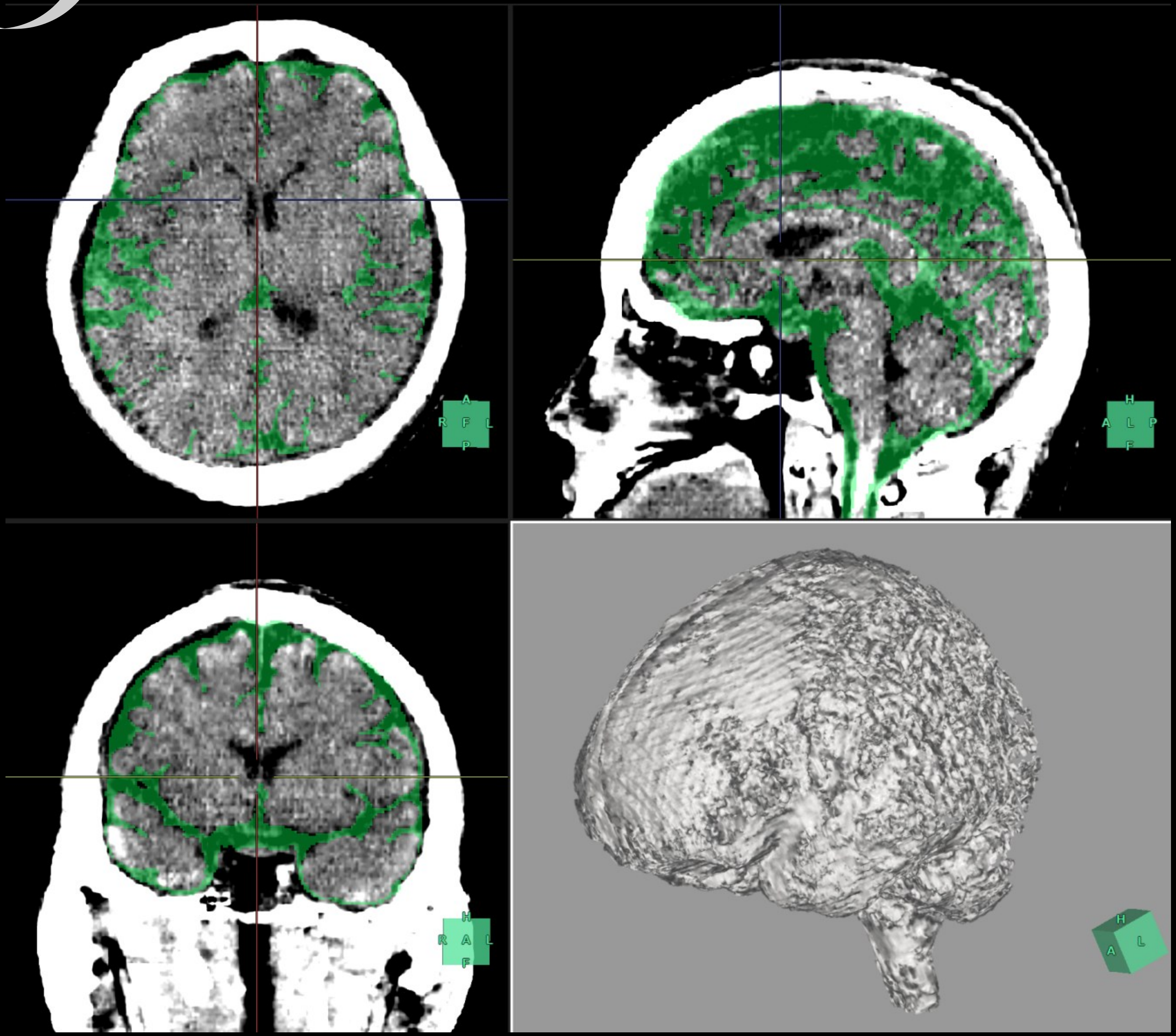

Normal (43y, Female) Total subarachnoid space
