# Supplementary material for: CT-based automatic segmentation of key CSF regions for detecting disproportionately enlarged subarachnoid space hydrocephalus
Source: Fluids Barriers CNS. 2026 Jun 23;23:83. doi: 10.1186/s12987-026-00814-5 (PMC13289119; doi:10.1186/s12987-026-00814-5)
Supplement: Supplementary file 2 — Supplementary Material 2 [file 12987_2026_814_MOESM2_ESM.pdf]

Semantic Segmentation (Version 1) with Synapse Creative Space → Manual Correction with Synapse Vincent 3D Viewer App

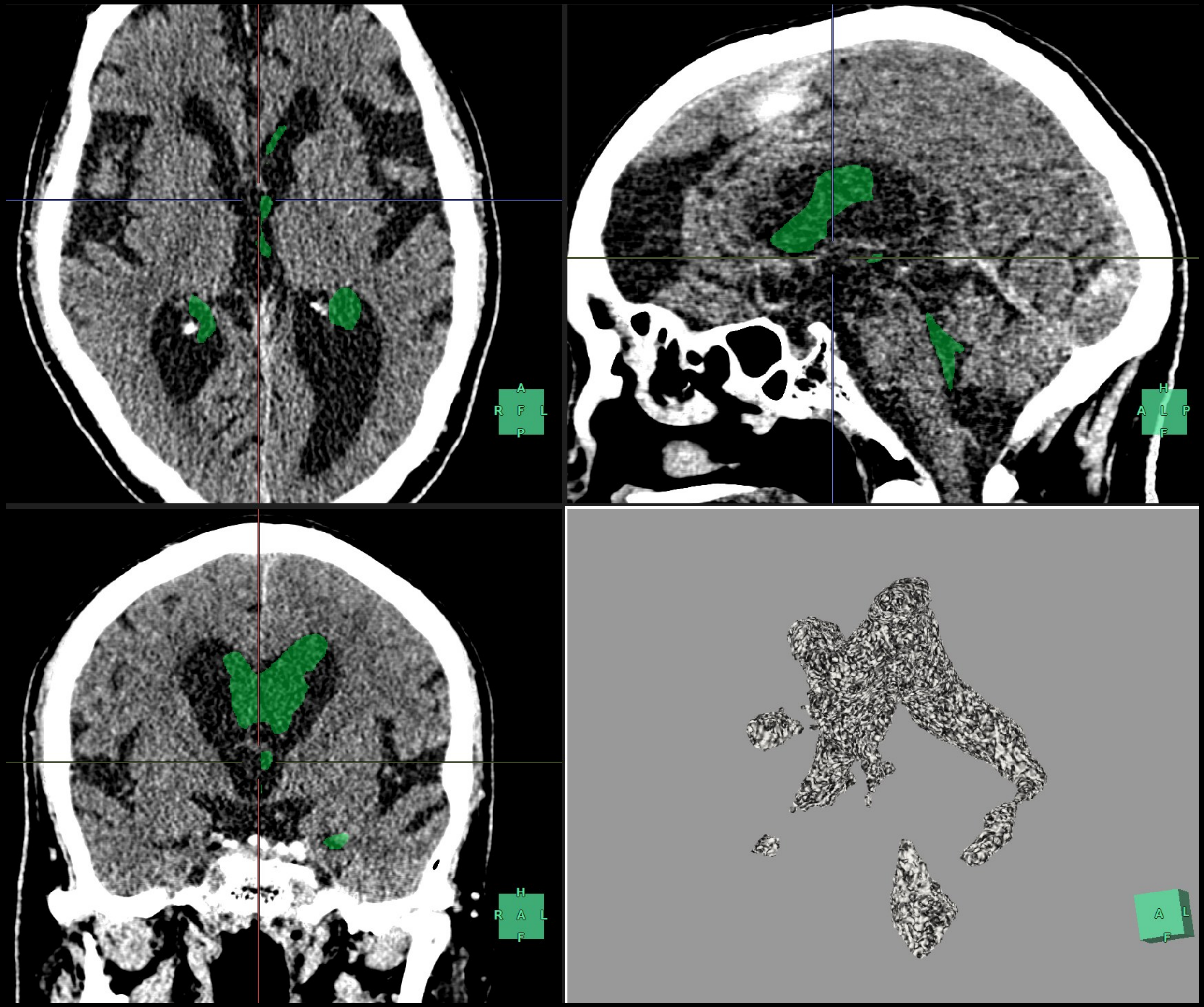

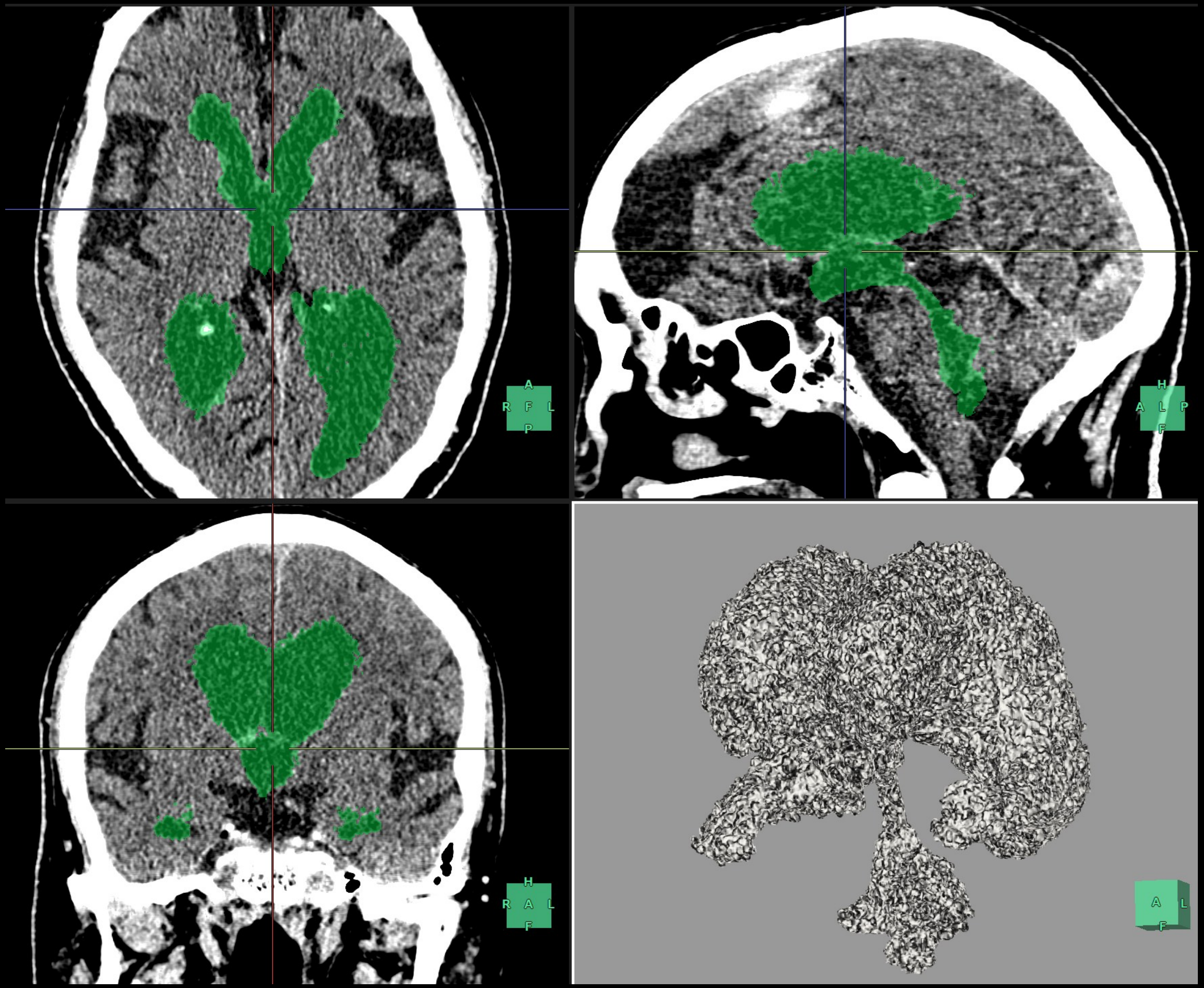

Hakim's disease (iNPH, 71y, Male) Total ventricle

(Thickness: 0.5 mm)

Segmentation (Version 1) ➡ Manual Correction

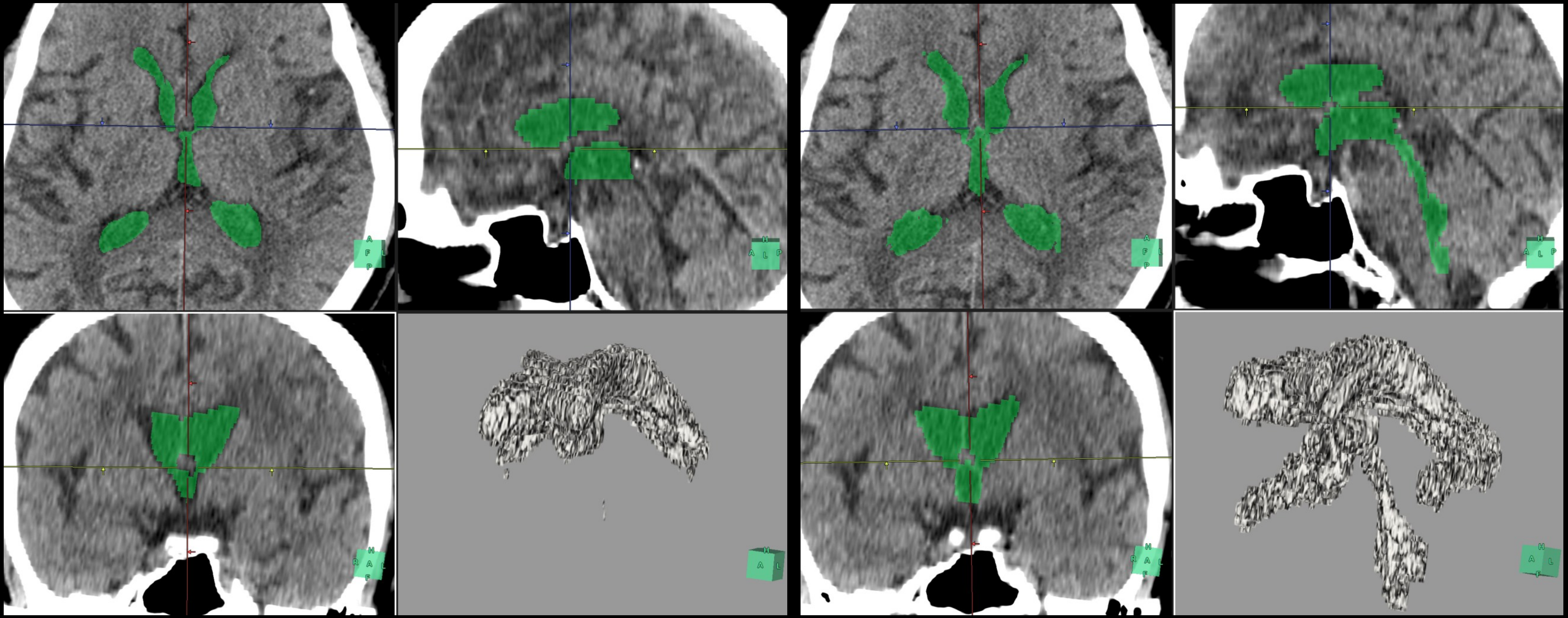

Normal (65y, Male) Total ventricle

(Thickness: 2.0 mm)

Segmentation (Version 1) ➡ Manual Correction

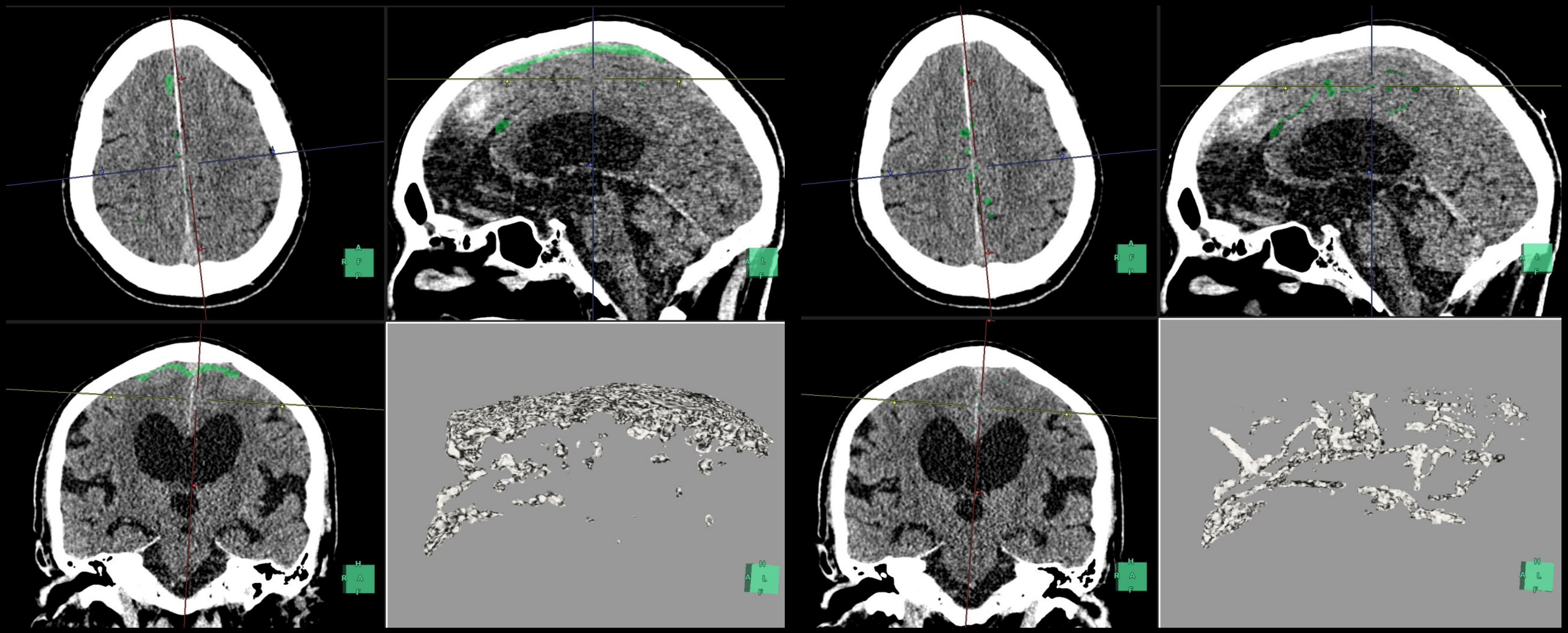

Hakim’s disease (iNPH, 71y, Male) High-convexity subarachnoid space (Thickness: 0.5 mm)

Segmentation (Version 1) ➡ Manual Correction

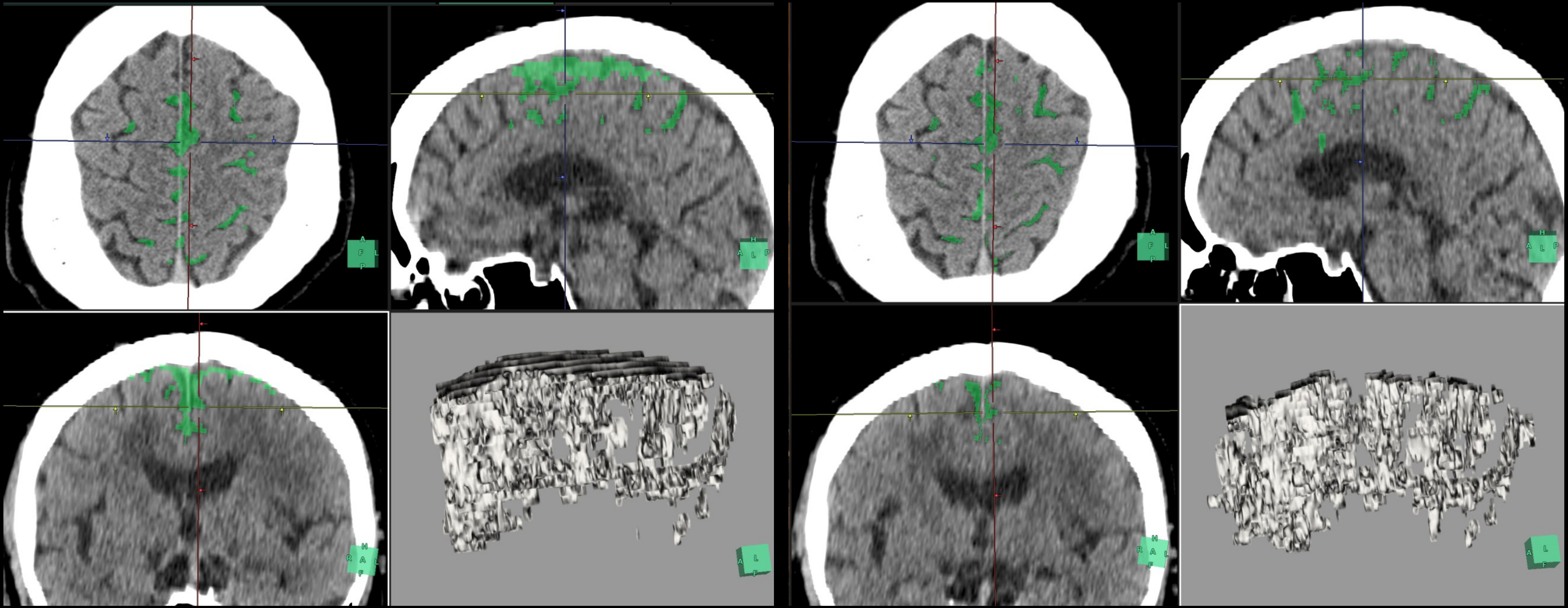

Normal (65y, Male) High-convexity subarachnoid space

(Thickness: 2.0 mm)

Segmentation (Version 1) ➡ Manual Correction

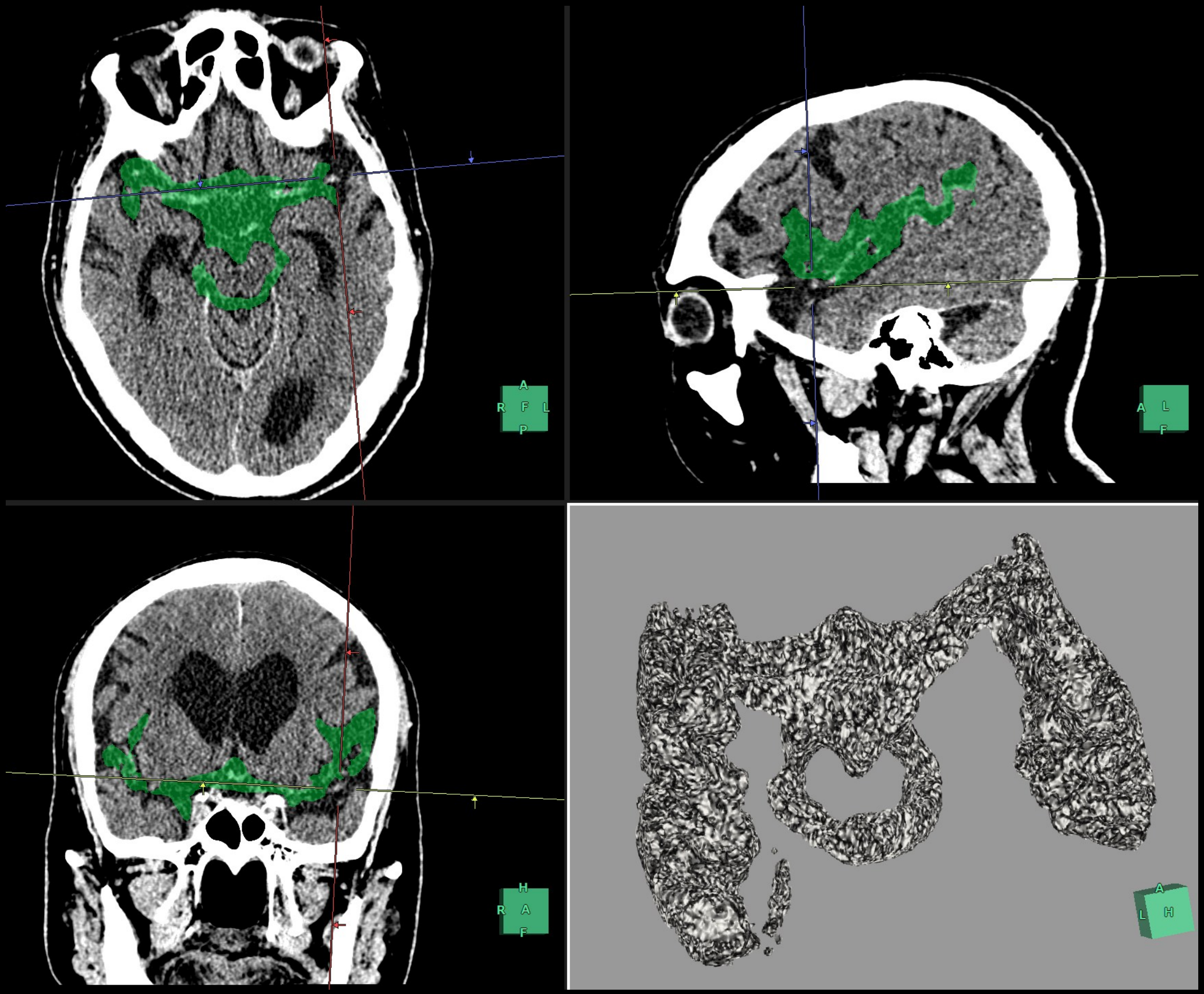

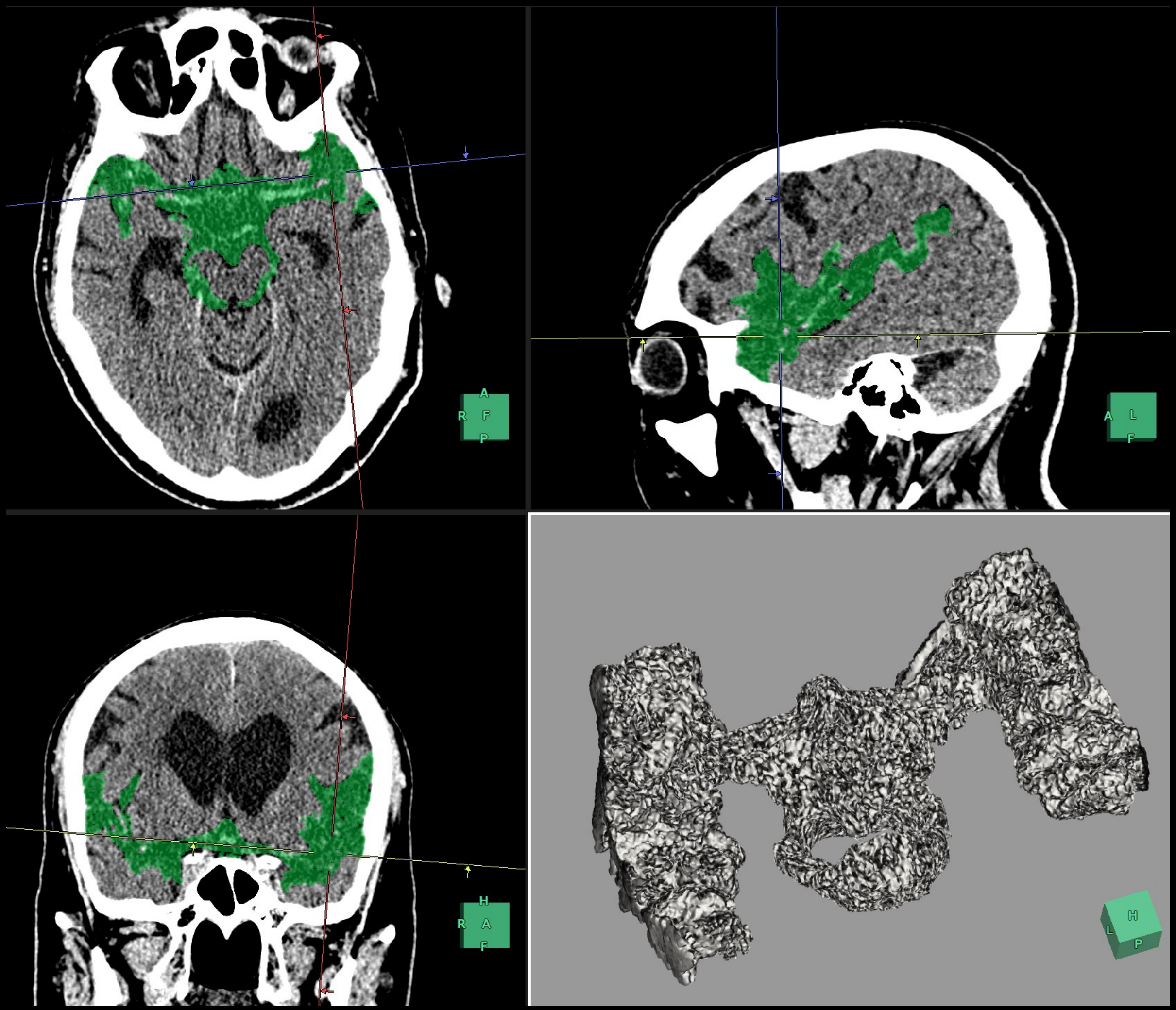

Hakim's disease (iNPH, 71y, Male) Sylvian fissure and basal cistern (Thickness: 0.5 mm)

Segmentation (Version 1) → Manual Correction

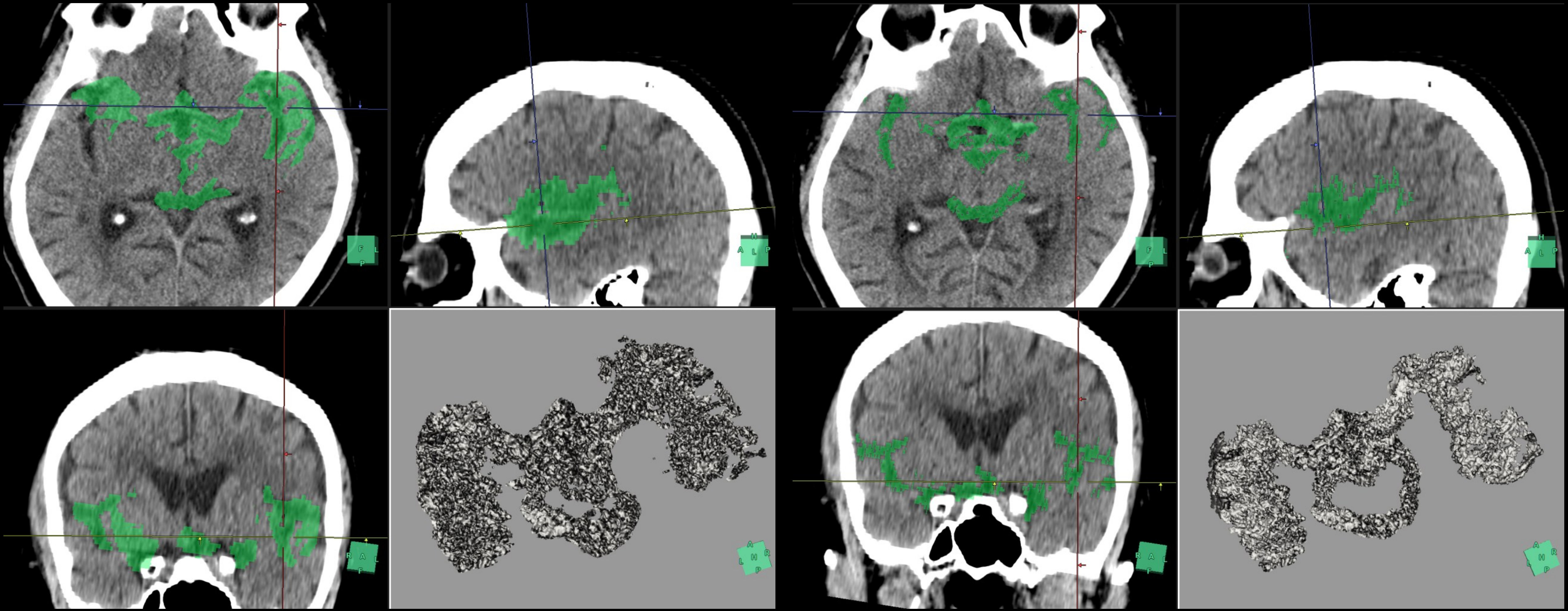

Normal (65y, Male) Sylvian fissure and basal cistern

(Thickness: 2.0 mm)

Segmentation (Version 1) ➡ Manual Correction

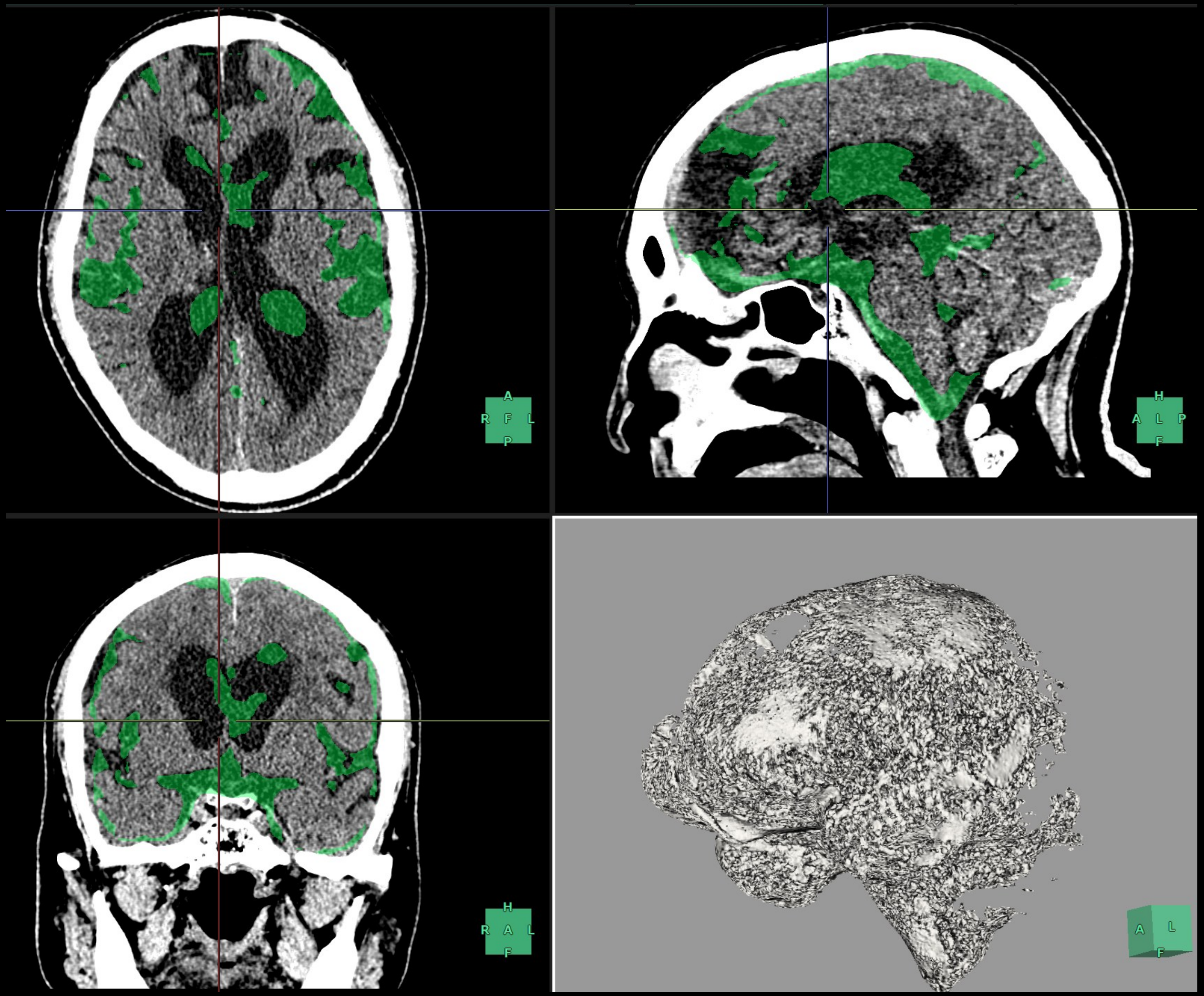

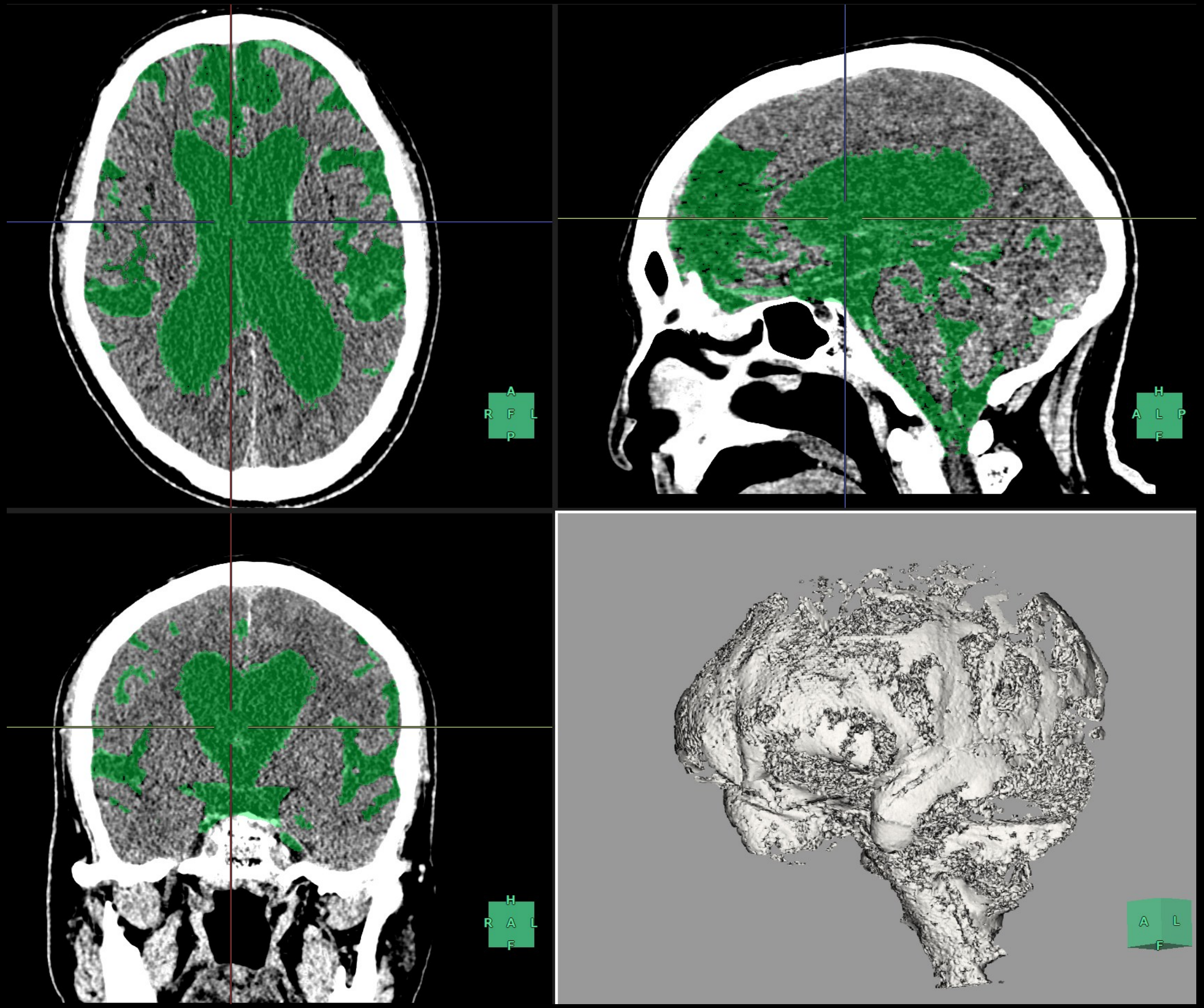

Hakim's disease (iNPH, 71y, Male) Total subarachnoid space (Thickness: 0.5 mm)

Segmentation (Version 1) ➡ Manual Correction

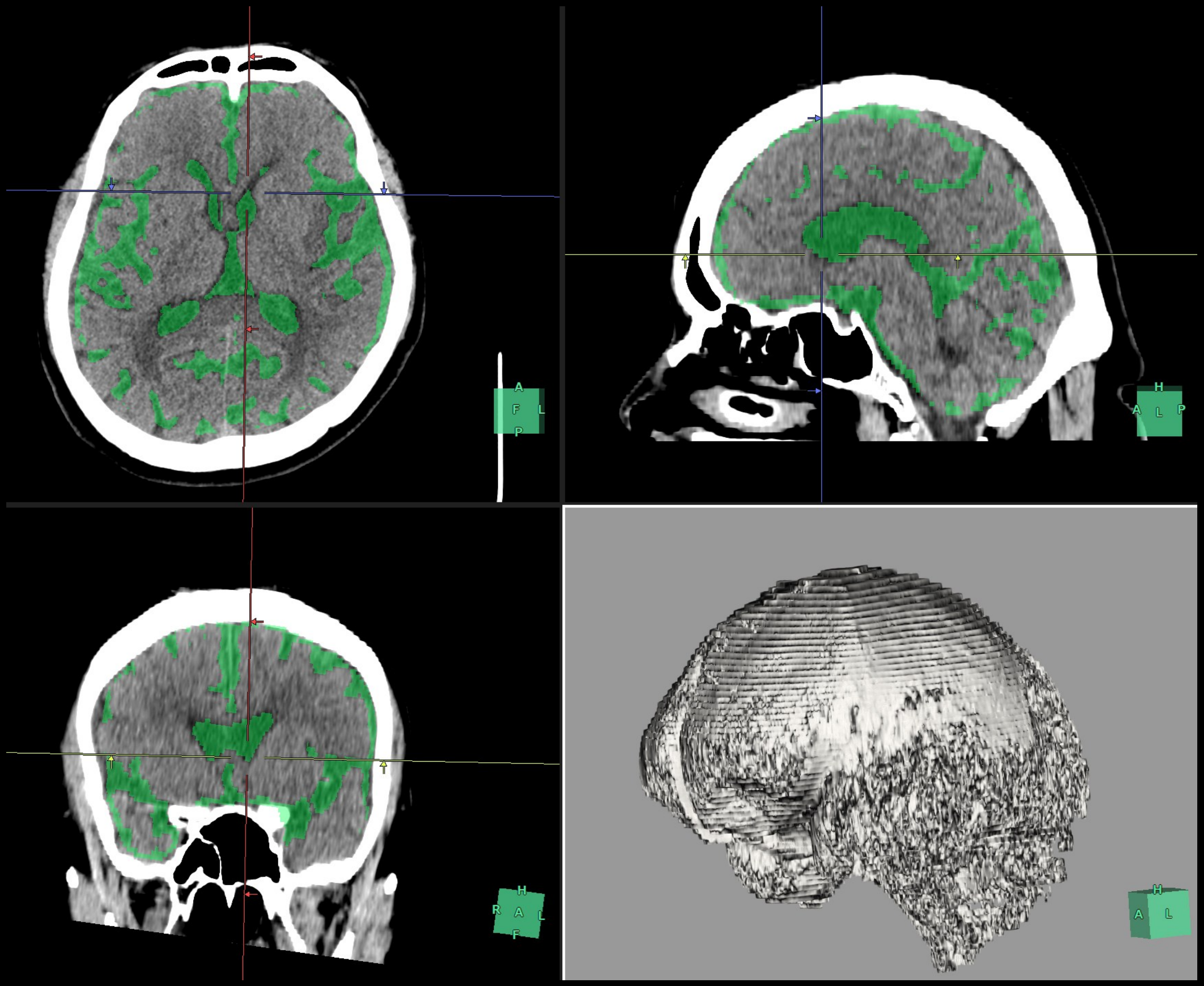

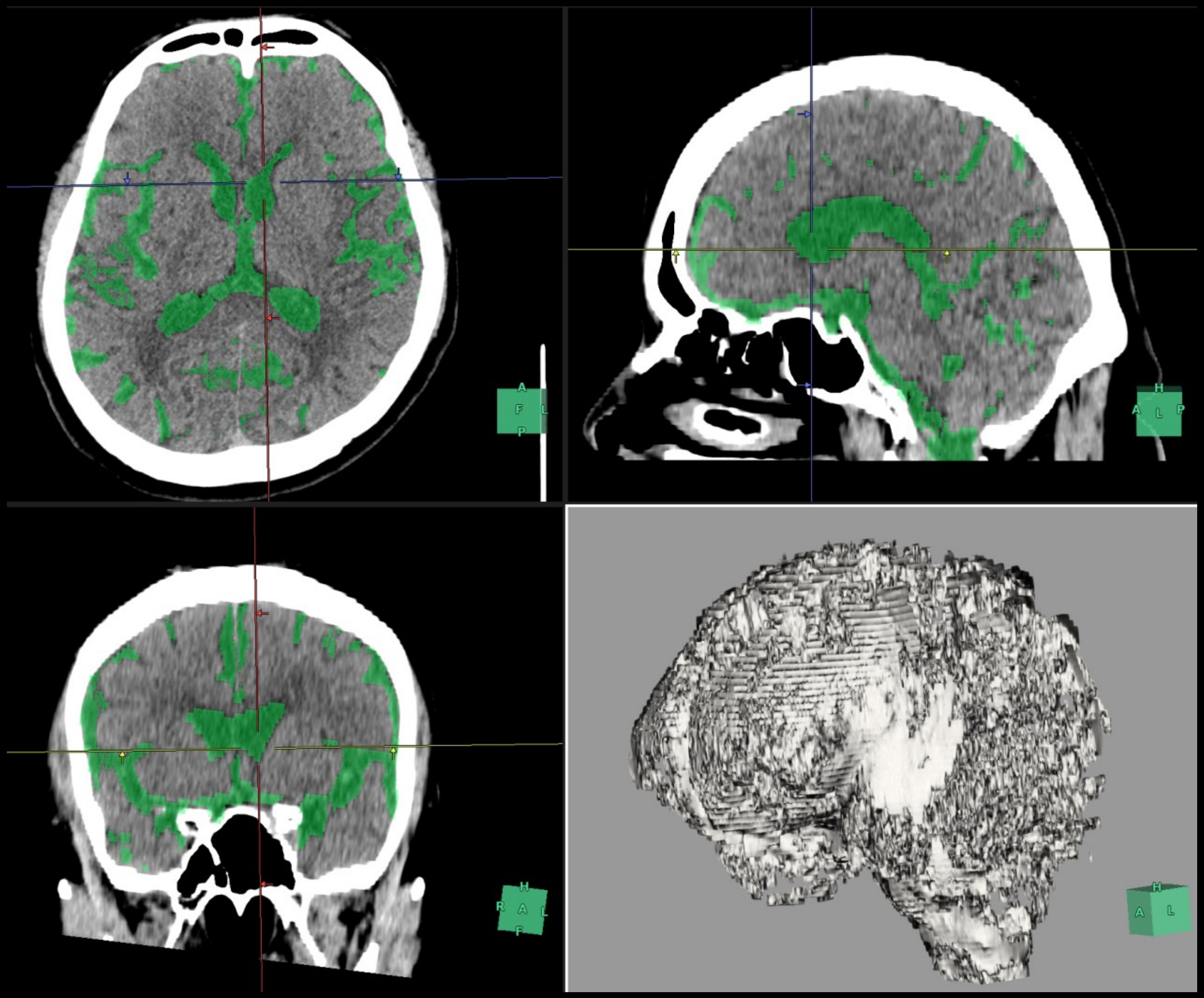

Normal (65y, Male) Total subarachnoid space

(Thickness: 2.0 mm)
