# Supplementary material for: CT-based automatic segmentation of key CSF regions for detecting disproportionately enlarged subarachnoid space hydrocephalus
Source: Fluids Barriers CNS. 2026 Jun 23;23:83. doi: 10.1186/s12987-026-00814-5 (PMC13289119; doi:10.1186/s12987-026-00814-5)
Supplement: Supplementary file 3 — Supplementary Material 3 [file 12987_2026_814_MOESM3_ESM.docx]

**Legends of Additional files**

**Additional file 1**

Synthetic CT images annotated with segmentation masks for the total ventricles, total subarachnoid space (SAS), high-convexity SAS, and a combined region of the Sylvian fissure and basal cistern were generated from T1-weighted 3D MRI with existing annotations using a cycle-GAN model.

**Additional file 2**

Segmentation masks for the total ventricles, total intracranial CSF space, high-convexity SAS, and a combined region of the Sylvian fissure and basal cistern were generated using the first-generation semantic segmentation model. However, this attempt failed, as all regions required substantial manual correction.
